# Estimating Infected Blacklegged Tick Encounters Among Outdoor Workers in Minnesota

**DOI:** 10.1007/s10393-025-01753-7

**Published:** 2025-09-18

**Authors:** Jacob Cassens, Scott Larson, Kristofer Keller, Bruce H. Alexander, Jeff B. Bender, Jonathan D. Oliver

**Affiliations:** 1 Division of Environmental Health Sciences, School of Public Health, University of Minnesota, Minneapolis, MN 55455 USA; 2 Metropolitan Mosquito Control District, Saint Paul, MN 55104 USA; 3 Department of Public Health and Environment, Washington County Public Health, Stillwater, MN 55082 USA

**Keywords:** *Ixodes scapularis*, blacklegged ticks, occupational health, tick-borne pathogen, disease risk, *Borrelia burgdorferi*, anaplasma phagocytophilum

## Abstract

Outdoor workers are at increased risk of tick-borne diseases, yet we poorly understand the interaction between occupational risk factors and worker behavior. This study integrates active tick surveillance with worker-reported survey data to assess how occupational behaviors, demographic characteristics, and tick-prevention knowledge influence exposure to infected ticks. We collected blacklegged ticks (*Ixodes scapularis*) from three Minnesota counties to determine the infection prevalence and density of infected ticks for *Borrelia burgdorferi* and *Anaplasma phagocytophilum*. Molecular surveillance was coupled with outdoor worker surveys that ascertained exposure characteristics to model individual-specific probabilities of encountering infected ticks during their job responsibilities. From May to July 2023–2024, 872 ticks were collected, where 45.6% (*n* = 398) were infected with *B. burgdorferi* and 7.2% (*n* = 78) were infected with *A. phagocytophilum*. Across both years, maximum infected tick densities peaked in Carlos Avery Wildlife Management Area at 0.80 (0.22 [IQR 0.16, 0.48]) per 100 m^2^, were intermediate in Lake Elmo Park Reserve at 0.35 (0.12 [IQR 0.05, 0.18]) per 100 m^2^, and lowest in Whitewater Wildlife Management Area at 0.25 (0.04 [IQR 0.02, 0.13]) per 100 m^2^. Forty-two survey responses revealed individual probabilities of encountering infected ticks ranged from ~ 5 to 65% for *B. burgdorferi* and ~ 0–25% for *A. phagocytophilum*. Our results suggest that outdoor workers have a high probability of encountering infected ticks through occupational exposure, which was marginally associated with demographic factors (e.g., age) and preventive behaviors (e.g., tick checks, repellent use). This study reports elevated *B. burgdorferi* infection prevalence from adult (62.1%) and nymphal (36.5%) blacklegged ticks within Minnesota.

**Supplementary Information:**

The online version contains supplementary material available at 10.1007/s10393-025-01753-7.

## Introduction

Vector-borne diseases pose significant and emerging threats to public health, driven by climate-induced shifts in vector distribution. Currently, vector-borne diseases account for 17% of all infectious diseases worldwide (WHO, 2024). Ticks are the primary vectors of concern in the USA, contributing to over 77% of vector-borne disease cases annually (Rosenberg et al., 2018). Lyme disease is the most common tick-borne illness in the USA, with an estimated 500,000 cases diagnosed annually (Kugeler et al., 2021). Other tick-borne diseases, including bacterial, protozoal, and viral diseases, are also increasing in incidence (Rosenberg et al., 2018). Collectively, these diseases impose an annual economic burden of approximately USD 900 million, excluding associated social costs (Hook et al., 2022). No human vaccines are currently available in the USA, and treatment depends on the timely administration of antimicrobials, underscoring the challenge of mitigating tick-borne diseases. Younger and older individuals are commonly considered high-risk populations (Kugeler et al., 2022), although other factors, such as occupational exposure, likely contribute to elevated risks, particularly for those whose job responsibilities involve frequent contact with tick habitats.

Blacklegged ticks thrive in closed-canopy forests with high humidity and low solar radiation (Cassens et al., 2023). Outdoor workers, who frequently perform tasks in these habitats during peak tick activity, are presumed to have heightened exposure to ticks and tick-borne pathogens. However, few studies have systematically assessed occupational risks, leading to uncertainty regarding the extent and distribution of tick-borne pathogen exposure among outdoor workers. Previous research suggests that high-exposure outdoor workers have elevated tick-borne pathogen seroprevalence and frequently encounter ticks but often discount their personal risk (Adjemian et al., 2012; Chmielewska-Badora et al., 2012; Cisak et al., 2012; De Keukeleire et al., 2017; Fingerle et al., 1997; Jahfari et al., 2014; Schotthoefer et al., 2020; van Charante et al., 1998). Despite this, tick encounters among outdoor workers remain understudied, highlighting the need for integrated surveillance and epidemiological approaches to examine the relationship between worker behavior and tick exposure.

Tick-borne pathogen surveillance relies on active and passive methods. Active surveillance involves systematically collecting ticks from the environment to assess species distribution and abundance (or density) and pathogen prevalence, yet is labor- and cost-intensive (CDC, 2019). Passive surveillance, which collects ticks submitted by the public or healthcare providers, is more cost-effective but less comprehensive and generalizable (Koffi et al., 2012; Ripoche et al., 2018). Health departments commonly employ both methods, depending upon the funding, with dedicated teams performing active surveillance as part of the CDC’s standardized tick surveillance program (Eisen et al., 2024; Foster et al., 2024). These efforts estimate tick abundance, infection prevalence, and the density of infected ticks, often used to calculate the entomological hazard index for assessing human-tick-pathogen encounter risks (Holcomb et al., 2023). However, these estimates overlook human behavior, assuming that tick density directly correlates with human exposure. This assumption may underestimate tick-borne disease risk and weaken the correlation between entomological hazards and disease incidence.

To address this gap, we integrated active and passive surveillance approaches to evaluate tick exposure among outdoor workers in Minnesota. We administered surveys to outdoor workers examining their knowledge, attitudes, and practices (KAP) related to tick prevention and combined these data with active tick collections in worker habitats. Using a passive sampling model developed by Hassett et al. (2022), we estimated the probability of encountering infected ticks among outdoor workers based on job-related exposure. Our objective was to assess the exposure risks of individual outdoor workers by leveraging person-level survey responses, hypothesizing that outdoor workers with elevated exposure to tick habitats are at greater risk of encountering infected ticks during their weekly job responsibilities.

## Materials and Methods

### Tick Collections

**Figure 1:**
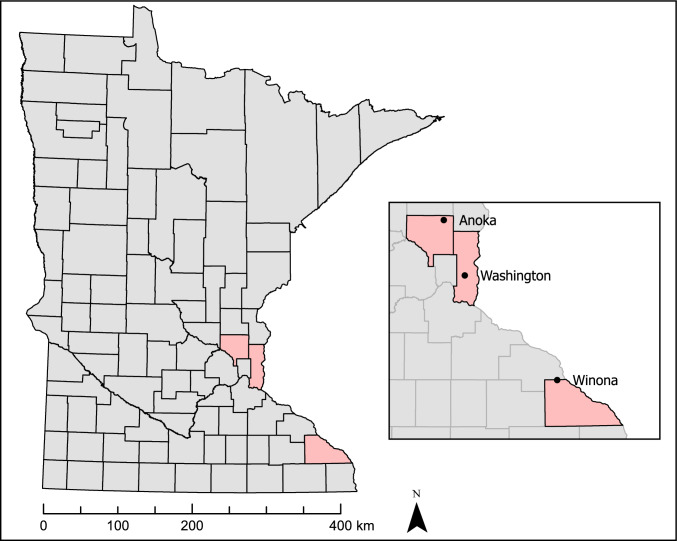
County-level map of Minnesota with black dots representing sampling locations for blacklegged tick collections. Sampling sites within each county were established for active tick collections, including Carlos Avery Wildlife Management Area (Anoka County, MN), Lake Elmo Park Reserve (Washington County, MN), and Whitewater Wildlife Management Area (Winona County, MN).

Tick collections were performed weekly from May 24th to July 11th to coincide with nymphal blacklegged tick activity patterns in Minnesota, as nymphs are deemed the most epidemiologically relevant life stage (Eisen and Eisen, 2018). Sampling transects were established at three sites frequented by outdoor workers throughout Minnesota. These included Lake Elmo Park Reserve (Washington County, MN), Carlos Avery Wildlife Management Area (Anoka County, MN), and Whitewater Wildlife Management Area (Winona County, MN) (Fig. 1). Transects were sampled by dragging a 1 m^2^ white flannel cloth across vegetation to collect actively questing ticks and inspected for attached ticks every 10 m. A minimum of 1000 m^2^ was dragged per visit. Drag lengths and sampling coordinates were monitored electronically using GPS Tracker Pro for iPhone v 1.8, allowing tick density estimation. Sampling effort was highest in Lake Elmo Park Reserve due to a dedicated tick team, whereas a single individual conducted collections in Whitewater WMA and Carlos Avery WMA. Sampling sites were visited at least five times per year, resulting in 92.9 km dragged in Lake Elmo Park Reserve, 5.2 km dragged in Carlos Avery WMA, and 17.2 km dragged in Whitewater WMA. Ticks were removed from the drag cloth using forceps, placed in 70% ethanol, and transported to the University of Minnesota for identification and molecular analysis. Ticks were morphologically identified to life stage using the taxonomic keys of Kierans and Clifford (1978) and Cooley and Kohls (1945).

### DNA Extraction and PCR

Total DNA was extracted from wild-caught blacklegged ticks using the Qiagen DNEasy Blood & Tissue kit (Qiagen, Hilden, Germany) following the manufacturer’s instructions. DNA extracts were quantified with a ThermoFisher Scientific NanoDrop One fluorometer and stored at − 20°C prior to PCR analysis. We leveraged two PCR assays to screen blacklegged ticks for *Borrelia burgdorferi* sensu stricto (Wills et al., 2018) and *Anaplasma phagocytophilum* (Goodman et al., 1996) using primers targeting OspA and 16S rRNA, respectively (Table 1). The *A. phagocytophilum* assay used in this study does not differentiate the human-active lineage (Ap-ha) from the non-human associated variant 1 lineage (Ap-V1). Amplification was performed in a Bio-Rad T100 Thermo Cycler (Bio-Rad Laboratories, Hercules, CA) following the PCR conditions reported in Wills et al. (2018) and Goodman et al. (1996), respectively. PCR reactions were performed in 25 µL solutions, with 0.5 µL each of 10 µM forward and reverse primers, 2 µL of template DNA, 12.5 µL of Taq 2*X* master mix, and 9.5 µL of nuclease-free water. Validated DNA isolates from our laboratory’s previous tick-borne pathogen surveillance were used as positive controls in respective PCR runs (Fountain‐Jones et al., 2023). PCR products were run on electrophoretic gels for one hour at 100 V and visualized with a 150-bp molecular ladder on a 2% agarose gel.

**Table 1:** PCR assay details for *Borrelia burgdorferi* and *Anaplasma phagocytophilum* detection.

Identification target	Primer name	Target gene	Sequence (5′–3′)	Amplicon size	References
*Anaplasma phagocytophilum*	PER1	16S rRNA	TTTATCGCTATTAGATGAGCCTATG	451 bp	Goodman et al., 1996
	PER2	16S rRNA	CTCTACACTAGGAATTCCGCTAT		
*Borrelia burgdorferi* s.s	OspA Out Fw	OspA	CTTGAAGTTTTCAAAGAAGAT	503 bp	Wills et al., 2018
	OspA Out Rv	OspA	CAACTGCTGACCCCTCTAAT	447 bp	
	OspA In Fw	OspA	ACAAGAGCAGACGGAACCAG	487 bp	
	OspA In Rv	OspA	TTGGTGCCATTTGAGTCGTA	350 bp	

*B. burgdorferi* detection leveraged a nested PCR assay, with the amplicons from the outer primers carried forward as template DNA for use with the inner primers. *A. phagocytophilum* assays were not capable of differentiating the human-active variant (Ap-ha).

### Knowledge, Attitudes, and Practices Survey

We administered KAP surveys to outdoor workers at two agencies that regularly conduct active tick collections in Minnesota, the Metropolitan Mosquito Control District and the Washington County Public Health and Environment. Participants were recruited through email distributions disseminated via collaborators, accompanied by a study description, a letter of support from their agency encouraging participation, and a link to participate. Participants were able to complete questionnaires on any available electronic device via Qualtrics (Provo, UT), where participants were required to review study procedures and grant consent before responding anonymously. Questionnaires were designed to minimize time requirements (~ 5 min) and based on previous KAP surveys administered to outdoor workers in the upper Midwest (Schotthoefer et al., 2020). Demographic questions included closed-ended ordered responses for age, gender, and employment history. Knowledge, attitudes, and practices questions included closed-ended ordered responses for tick check frequency, frequency of finding ticks on oneself, repellent usage, concern of tick-borne diseases, and history of tick-borne disease diagnosis. Two open-ended free-response questions were designed to ascertain (1) self-reported exposure to tick habitats as part of their job responsibilities during summer months and (2) how much time an individual spent working per week during summer months. An individual’s self-reported exposure to tick habitat per week was divided by their self-reported hours working per week to estimate the proportion of their working hours spent in tick habitat to parameterize a passive sampling model described below. Full questionnaires can be found in Supplementary File S1.

### Data Analysis

#### Tick and Tick-Borne Pathogen Analysis

Tick counts were collated and divided by the GPS-tracked sampling transect distance to obtain the density of nymphs, adults, and total ticks for each site by the year of collection. The density of nymphs, adults, and total ticks for each site and year were then standardized to 100 m. Tick infection prevalence estimates were calculated for *B. burgdorferi* and *A. phagocytophilum* by dividing the number of infected nymphs, adults, and total ticks by the respective tick count for each site by the year of collection. These estimates were multiplied (infection prevalence *x* density of ticks) to compute the entomological hazard index, representing the density of infected nymphs, adults, and total ticks for each site by the year of collection and pathogen. The entomological hazard index was then used to parameterize a passive sampling model described below. Alternatively, to validate the density of infected nymphs, adults, and total ticks calculated using this approach, the number of infected nymphs, adults, and total ticks was divided by the standardized transect distance per site per year.

#### Probability of Infected Tick-Human Encounter

We extended the methodology developed by Hassett et al. (2022), which estimates the cumulative probability of encountering ticks, to evaluate the cumulative probability of encountering infected blacklegged ticks among outdoor workers during their job responsibilities. To do so, we utilized the passive sampling model formulated by Hassett et al. (2022) and briefly described here. The passive sampling method assumes that the probability of encountering a species (e.g., tick) in a defined area (e.g., tick habitat) depends on the time spent in that area and the number of randomly distributed individuals of that species (Gotelli and Graves, 1996). Under this scenario, we can estimate the probability of a human-tick encounter by targeting sampling efforts to blacklegged tick habitat frequented by outdoor workers, representing the defined area, the time spent in tick habitat as the self-reported exposure during job responsibilities, and infected tick density as the number of randomly distributed individuals of blacklegged ticks. We assumed that the probability of an outdoor worker encountering an infected tick while working in a tick habitat increases with the time spent in tick habitat:

$$ P({\mathrm{tick}}) = 1 - \left( {1 - \Delta t/P} \right) $$

where delta *t* represents an individual’s self-reported exposure to tick habitat (hours) as part of their weekly job responsibilities, and *P* represents an individual’s self-reported weekly working schedule (hours), ascertained through outdoor worker survey responses. However, our primary interest is the probability of encountering any infected tick while in tick habitat. As such, the probability of encountering infected ticks can be formulated as:

$$ P({\mathrm{tick}}) = 1 - \left( {1 - \Delta t/P} \right)^{n} $$

where *n* represents the number of infected ticks. Yet, we are interested in the number of randomly distributed species rather than the crude number of infected ticks. Therefore, we can replace the number of infected ticks (*n*) with the density of infected ticks (*d*; per 100 m^2^) to obtain the probability of encountering an infected tick by an outdoor worker as part of their job responsibilities per week:

$$ P({\mathrm{tick}}) = 1 - \left( {1 - \Delta t/P} \right)^{d} $$

This probability was estimated for each outdoor worker and tick life stage, although the predicted probability of encountering an infected tick of any life stage is reported here. Further, the predicted probability was calculated using the density of ticks for each year and the total over both years, with the latter being reported. Detailed information on models using specific life stages for either year is available in Supplementary File S2.

All analyses were performed in R (R Core Team, 2022). R code for the analysis can be found in Supplementary File S3.

#### Knowledge, Attitudes, and Practices Responses

KAP responses were downloaded from Qualtrics and summarized using tableone (Yoshida and Bartel, 2014). Individuals who did not consent to participate but completed the survey were removed from the analysis. Categorical variables were re-leveled to ensure the appropriate reference category was used for comparisons. Self-reported exposure to tick habitat was dichotomized using the median value of their numerical response to describe potential variability between groups. Two complementary analytical approaches were employed to examine different aspects of tick exposure risk among outdoor workers. First, to assess how worker characteristics and knowledge influence self-reported exposure behaviors, ordinal regression models were run using MASS (Ripley and Venables, 2009) to determine associations between individuals’ self-reported exposure and KAP responses, adjusting for gender and age (Supplementary Table S1). Mixed effects from regression models were obtained using ggeffects (Lüdecke, 2018). Multinomial models were parameterized and compared with ordinal regression models for each KAP response. Ordinal regression models had lower AIC values and were chosen for downstream analysis. Second, to explore whether individual knowledge, attitudes, and practices are associated with estimated infected tick encounter probabilities, generalized linear regression models (GLMs) were run using the stats package in R to evaluate the association between outdoor worker KAP responses and their estimated predicted probability of encountering an infected tick. This exploratory analysis aimed to identify individual-level factors that may contribute to variation in occupational tick encounter risks, beyond direct exposure time. Models were both (1) stratified by location to account for site-specific differences in work environments and tick ecology that may influence exposure patterns (Supplementary Table S2) and (2) averaged across all three sites for comparison (Supplementary Table S6). Estimated predicted probabilities for each site and averaged overall three sites were used as dependent variables in independent regression models. Potential multicollinearity among predictors (age, gender, length of employment, etc.) were assessed using variance inflation factors, with values < 4 considered acceptable. To avoid multicollinearity, full models excluded workers’ self-reported exposure time and work hours per week, as these variables were used to calculate infected tick encounter probabilities and had variance inflation factors > 4. Model coefficients were exponentiated to obtain odds ratios and their respective 95% confidence intervals for interpretation. Forest plots for regression results were created using forest plot (Gordon and Lumley, 2014) and ggplot2 (Wickham, 2016).

Three modeling approaches were implemented to evaluate associations between KAP responses and estimated predicted probabilities: (1) full models with all KAP responses (Supplementary Tables S2 and S6); (2) stepwise model selection (ΔAIC > 4; Supplementary Tables S3 and S7); (3) multi-model inference using the MuMin (Kamil Bartoń, 2010; Supplementary Tables S4 and S8). Stepwise selection retained only tick check frequency and age, whereas approaches (1) and (3) provided nearly identical model results. We are primarily interested in understanding KAP relationships rather than prediction, and therefore, the full model results from approach (1) are reported (Tables 4 and 5). Model results are presented across all sites, with results (Supplementary Tables S2–S4 and S6–S8) and mixed effects (Supplementary Tables S5 and S9) from (2) to (3) available in Supplementary File S2.

## Results

### Tick Summary Stats

Tick collections from late May to early July in 2023 and 2024 totaled 115,292 m^2^ dragged across three sites displayed in Fig. 1. A total of 872 ticks were collected, comprising 64% nymphs (*n* = 561) and 36% adults (*n* = 311). Notably, 80% of adults (*n* = 249) were collected in Lake Elmo Park Reserve in 2023, and 68% of nymphs (*n* = 382) were also from this site, reflecting the greater sampling effort. Across all sites, substantially more adults were collected in 2023 (*n* = 277) than in 2024 (*n* = 34), while nymph numbers remained relatively constant in 2023 (*n* = 271) and 2024 (*n* = 290). Tick densities varied by location, ranging from 0.3 to 0.71 per 100 m^2^ in Lake Elmo Park Reserve, 0.47–2.7 per 100 m^2^ in Carlos Avery WMA, and 0.07–0.44 per 100 m^2^ in Whitewater WMA (Table 2).

**Table 2:** Blacklegged tick counts and density (per 100 m^2^) for total and infected nymphs, adults, and all ticks at each sampling site in 2023, 2024, and the total across both years.

County	Site	Habitat	Forest type	Drag distance (m)	Ticks collected			*Borrelia burgdorferi*			*Anaplasma phagocytophilum*		
					Nymphs	Adults	Total	Nymphs	Adults	Total	Nymphs	Adults	Total
*2023*													
Washington	Lake Elmo Park Reserve	Trail	Mixed	64,000	189 (0.3)	249 (0.39)	438 (0.68)	89 (0.14)	156 (0.24)	245 (0.38)	16 (0.03)	28 (0.04)	44 (0.07)
Anoka	Carlos Avery WMA	Edge	Deciduous	2983	47 (1.6)	17 (0.57)	64 (2.15)	9 (0.3)	9 (0.3)	18 (0.6)	3 (0.1)	3 (0.1)	6 (0.2)
Winona	Whitewater WMA	Trail	Mixed	8846	35 (0.4)	11 (0.12)	46 (0.52)	14 (0.16)	8 (0.09)	22 (0.25)	2 (0.02)	1 (0.01)	3 (0.03)
*2024*													
Washington	Lake Elmo Park Reserve	Trail	Mixed	28,923	193 (0.67)	26 (0.09)	219 (0.76)	61 (0.21)	16 (0.06)	77 (0.27)	14 (0.05)	5 (0.02)	19 (0.07)
Anoka	Carlos Avery WMA	Edge	Deciduous	2170	68 (3.1)	7 (0.32)	75 (3.5)	20 (0.92)	3 (0.14)	23 (1.1)	4 (0.18)	1 (0.05)	5 (0.23)
Winona	Whitewater WMA	Trail	Mixed	8370	29 (0.35)	1 (0.01)	30 (0.36)	12 (0.14)	1 (0.01)	13 (0.16)	1 (0.01)	0 (0.00)	1 (0.01)
*Total*													
Washington	Lake Elmo Park Reserve	Trail	Mixed	92,923	382 (0.41)	275 (0.30)	657 (0.71)	150 (0.16)	172 (0.19)	322 (0.35)	30 (0.03)	33 (0.04)	63 (0.07)
Anoka	Carlos Avery WMA	Edge	Deciduous	5153	115 (2.2)	24 (0.47)	139 (2.7)	29 (0.56)	12 (0.23)	41 (0.80)	7 (0.14)	4 (0.08)	11 (0.21)
Winona	Whitewater WMA	Trail	Mixed	17,216	64 (0.37)	12 (0.07)	76 (0.44)	26 (0.15)	9 (0.05)	35 (0.20)	3 (0.02)	1 (0.01)	4 (0.02)

Each sampling site was visited at least five times per year. Tick drags were performed using a 1 m^2^ drag cloth and drag distances were monitored electronically using GPS Tracker Pro.

**Figure 2:**
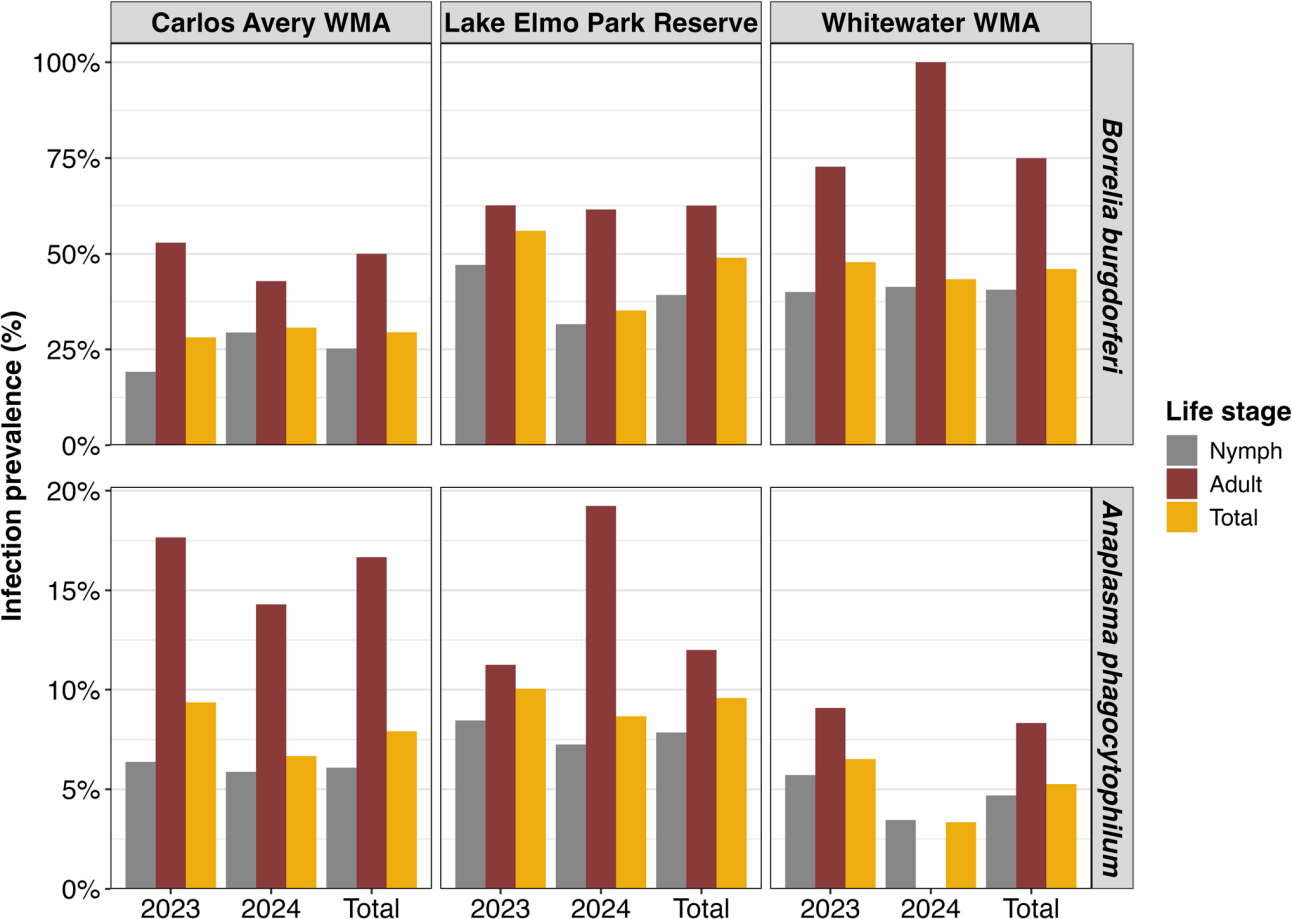
Infection prevalence estimates for *Borrelia burgdorferi* and *Anaplasma phagocytophilum* among nymphs, adults, and total ticks by site and year. Sample sizes for each category are available in Table 2, under total ticks collected for each life stage and total, respectively. The elevated *B. burgdorferi* infection prevalence for Whitewater WMA stems from low sample sizes, with one adult tested and infected in 2024.

PCR analysis assessed the presence of *B. burgdorferi* and *A. phagocytophilum* in collected ticks, enabling calculation of pathogen prevalence and the density of infected ticks. In total, 398 ticks were infected with *B. burgdorferi* and 63 with *A. phagocytophilum* (Table 2). Infection prevalence was highest in adults across all sites and years, consistent with the additional blood meal taken. Carlos Avery WMA had the highest adult prevalence for *B. burgdorferi*, but low adult tick counts in 2023 (*n* = 11) and 2024 (*n* = 1) limit confidence in these estimates. Lake Elmo Park Reserve consistently showed high *B. burgdorferi* prevalence, reaching 63% in adults and 39% in nymphs (Fig. 2), among the highest recorded in Minnesota. Similarly, Carlos Avery WMA showed 50% adult prevalence and 25% nymphal prevalence, despite a low sample size (Fig. 2). Whitewater WMA nymphal infection prevalence was 41%, although limited sample sizes for nymphs and adults limit the interpretability of these results. In contrast, *A. phagocytophilum* prevalence was lower, with Lake Elmo Park Reserve reporting the highest numbers of infected nymphs (*n* = 30) and adults (*n* = 33), with prevalence ranging from 8 to 12% (Table 2). Although Carlos Avery WMA showed the highest *A. phagocytophilum* prevalence, low adult counts (*n* = 24) created substantial uncertainty. The density of infected ticks mirrored overall tick densities, highest in Carlos Avery WMA, followed by Lake Elmo Park Reserve and Whitewater WMA (Table 2).

### Outdoor Worker Exposure Summary Stats

Surveys were administered to outdoor workers employed by Washington County and the Metropolitan Mosquito Control District using Qualtrics throughout the study period. The twelve-question survey assessed demographic characteristics and workers’ knowledge, attitudes, and practices regarding tick exposure. In total, 62 responses were received, with 15 respondents (24.2%) not consenting to participate, and thus excluded from analysis, leaving 47 consenting responses (75.8%) for evaluation, with equal representation of males and females (*n* = 21 each) and fewer non-binary respondents (*n* = 5). Most respondents were under 25 or between 25 and 39 years old (Table 3). Nearly, all participants reported performing tick checks during their job responsibilities, although many rarely or never found ticks on themselves. Interestingly, the majority of respondents reported infrequent use of insect repellents (e.g., sometimes), and over one-fourth were not concerned about acquiring a tick-borne disease (Table 3). Respondents were dichotomized by their median self-reported exposure to tick habitats to partition exposure patterns into high and low-tick habitat exposure groups. Those in the high-exposure group reported spending an average of 34 h per week in tick habitat, compared to 20 h for those in the low-exposure group (Table 3). The proportion of individuals performing daily tick checks was similar between the high- (61.5%) and low-exposure groups (52.9%). Individuals in the high-exposure category reported always (53.8%) or sometimes (46.2%) using insect repellants, whereas a lower proportion of individuals reported always using insect repellants in the low-exposure category (26.5%).

**Table 3:** Outdoor worker responses to administered questionnaires.

Sample characteristic	Low exposure to tick habitat (*n* = 34)	High exposure to tick habitat (*n* = 13)	Total (*n* = 47)
Demographics			
Gender (%)			
Male	16 (47.1)	5 (38.5)	21 (44.7)
Female	15 (44.1)	6 (46.2)	21 (44.7)
Non-binary/third gender	3 (8.8)	2 (15.4)	5 (10.6)
Age (%)			
< 25	18 (52.9)	6 (46.2)	24 (51.1)
25–39	11 (32.4)	4 (30.8)	15 (31.9)
40–55	4 (11.8)	1 (7.7)	5 (10.6)
> 55	1 (2.9)	2 (15.4)	3 (6.4)
Occupational characteristics			
Employed in current role (%)			
< 1 year	7 (20.6)	4 (30.8)	11 (23.4)
1–2 years	7 (20.6)	4 (30.8)	11 (23.4)
> 2 years	20 (58.8)	5 (38.5)	25 (53.2)
Hours spent working in tick habitat (mean (SD))	20.0 (9.8)	34.4 (4.4)	24.0 (10.8)
Insect repellant use (%)			
Always	9 (26.5)	7 (53.8)	16 (34.0)
Sometimes	21 (61.8)	6 (46.2)	27 (57.4)
Never	4 (11.8)	0 (0.0)	4 (8.5)
Ticks found on oneself (%)			
0 times per week	9 (26.5)	5 (38.5)	14 (29.8)
1–2 times per week	21 (61.8)	8 (61.5)	29 (61.7)
> 2 times per week	4 (11.8)	0 (0.0)	4 (8.5)
Tick check frequency (%)			
Every day	11 (32.4)	8 (61.5)	19 (40.4)
Multiple times a day	7 (20.6)	0 (0.0)	7 (14.9)
Some days	10 (29.4)	3 (23.1)	13 (27.7)
Only when in tick habitat	6 (17.6)	1 (7.7)	7 (14.9)
Never	0 (0.0)	1 (7.7)	1 (2.1)
Concern about acquiring tick-borne disease (%)			
Extremely	4 (11.8)	4 (30.8)	8 (17.0)
Moderately	7 (20.6)	1 (7.7)	8 (17.0)
Somewhat	14 (41.2)	5 (38.5)	19 (40.4)
Not at all	9 (26.5)	3 (23.1)	12 (25.5)
History of tick-borne disease diagnosis (%)			
No	33 (97.1)	12 (92.3)	45 (95.7)
Yes	1 (2.9)	1 (7.7)	2 (4.3)

Self-reported exposure to tick habitat was dichotomized using the median value of their numerical response to describe potential variability between groups. Surveys were administered electronically using Qualtrics and required consent for inclusion into the study.

### Probability of Human and Infected Tick Encounters

**Figure 3:**
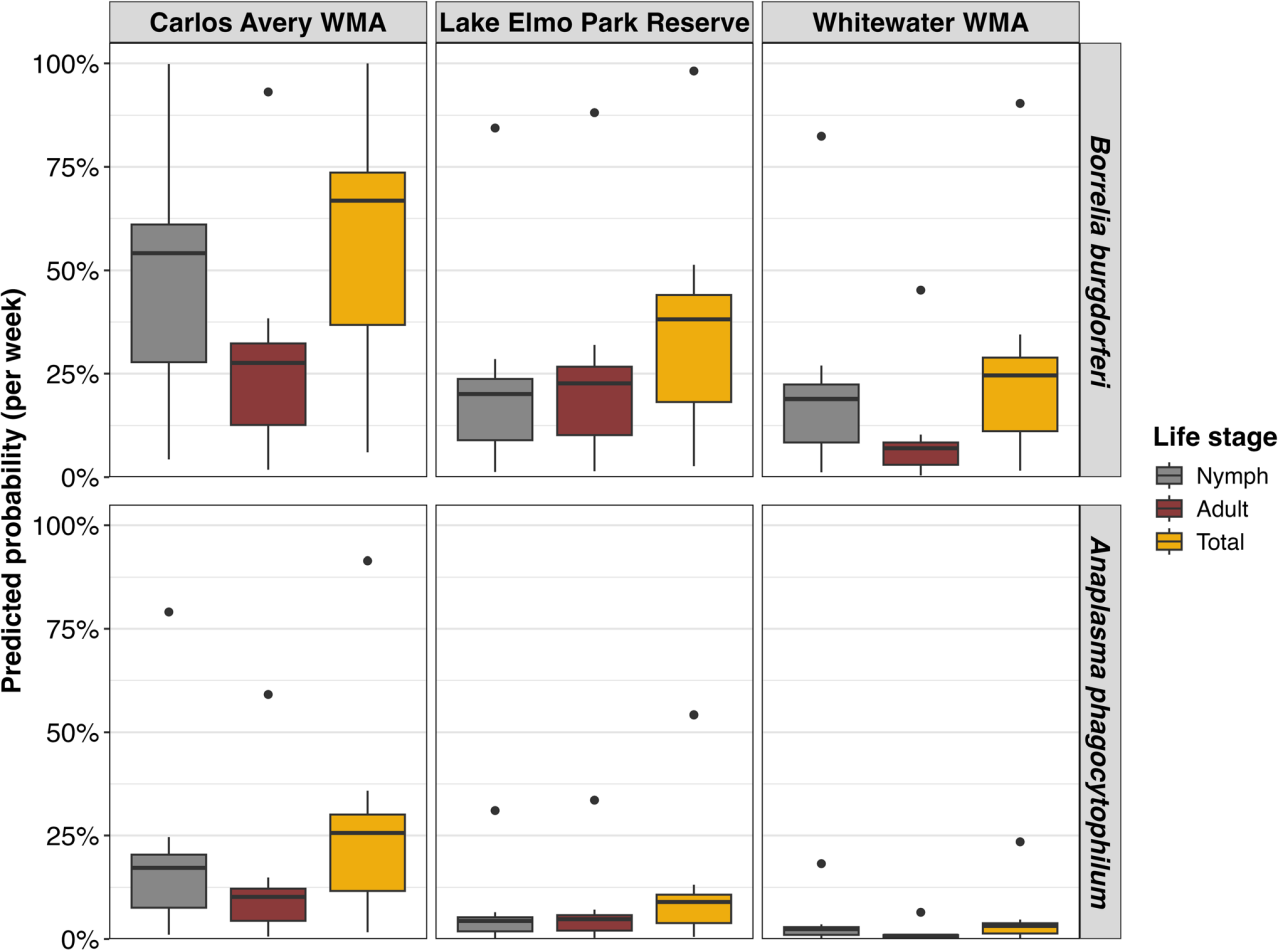
Distribution of outdoor workers predicted infected tick encounter probabilities for each site and tick life stage, computed using a passive distribution model parameterized with the proportion of time spent in tick habitats during job responsibilities from outdoor worker surveys. Predicted probability distributions are separated by pathogen and site for nymphal, adult, and total ticks.

We estimated the predicted probability of outdoor workers encountering infected ticks during their weekly job responsibilities by combining self-reported exposure time in tick habitats with the entomological hazard index (i.e., density of infected ticks). Exposure was calculated as the proportion of time spent in tick habitats during an average work week. Predicted probability estimates varied considerably across sites and tick life stages. In Carlos Avery WMA, outdoor workers had the highest predicted weekly probability (46% [IQR 0.28–0.61]) of encountering a *B. burgdorferi*-infected nymph, decreasing to 27% (IQR 0.13–0.32) for adults and 56% (IQR 0.37–0.74) for total ticks (Fig. 3). In contrast, the predicted probability of encountering any infected tick was lower in Lake Elmo Park Reserve (35% [IQR 0.18–0.44]) and lowest in Whitewater WMA (25% [IQR 0.11–0.29]). Due to the low infection prevalence of *A. phagocytophilum*, the estimated probability of encountering an infected tick was substantially lower across all sites and life stages. However, workers in Carlos Avery WMA had an estimated 26% (IQR 0.12–0.30) predicted probability of encountering an *A. phagocytophilum*-infected tick per week (Fig. 3). Whitewater WMA consistently showed the lowest predicted probabilities for both pathogens, reflecting lower tick counts, infection prevalences, and densities. However, it is important to note that our *A. phagocytophilum* PCR assay does not differentiate Ap-ha from Ap-V1, and thus, the estimated probability of encountering *Ap-*infected ticks is an overestimate of human anaplasmosis disease risk.

### Outdoor Worker Questionnaire Responses Summary

We used generalized linear regression models to assess the associations between outdoor workers estimated predicted probability of encountering an infected tick and their self-reported KAP responses, adjusting for age and gender. Across all sites, the odds of encountering a *B. burgdorferi* or *A. phagocytophilum-*infected tick were similar among all genders, holding all other variables constant (Tables 4 and 5). However, it is important to note that most associations in our analysis did not reach conventional statistical significance thresholds (*p* > 0.05), and confidence intervals for many estimates include the null value (1.00), indicating uncertainty about the direction and magnitude of these associations. Further, across all sites, outdoor workers aged 55 and older had higher odds of encountering an infected tick compared to younger age groups, with odds 39% greater for *B. burgdorferi* (1.39 [95% CI 1.00, 1.93]) and 32% greater for *A. phagocytophilum* (1.32 [95% CI 1.10, 1.59]) (Tables 4 and 5). For *B. burgdorferi*, this represents a marginal association that does not meet conventional significance thresholds. Although these odds ratios are elevated, the limited sample size among outdoor workers aged 55 and older (*n* = 3) limits our confidence in these estimates and should be interpreted with caution. Outdoor workers who reported consistent repellent use and finding ticks on themselves were associated with higher odds of encountering an infected tick, regardless of pathogen or site, controlling for other factors (Tables 4 and 5). Performing tick checks some days or every day was associated with increased odds of encountering a *B. burgdorferi*-infected tick across all sites, yet performing tick checks multiple times a day was associated with an odds reduction (Table 4). Performing any tick checks was associated with a reduction in the odds of encountering an *A. phagocytophilum*-infected tick (Table 5). We investigated this association by regressing self-reported tick check frequency against the absolute value of self-reported exposure to tick habitat (hours). We found that individuals who performed tick checks were more likely to report lower exposure, which contributed to the associations described above. Finally, being employed in your current role for more than two years was associated with a reduction in the odds of encountering an infected tick across all sites (Tables 4 and 5). These findings should be considered preliminary given our sample size constraints, and future studies with larger sample sizes are needed to definitively establish these associations. Subsequent regression analyses were stratified by site and year, demonstrating qualitative robustness with the models across all sites reported above, and results from these analyses are available in Supplementary File S2 (Tables S2 and S6).

**Table 4:** Full model results for generalized linear regression models evaluating the association between an individual’s predicted probability of encountering *Borrelia burgdorferi*-infected ticks and their survey responses averaged across all sampling sites.

	Estimate	SE	*p*-value	Odds ratio	95% CI
*All sites*					
Intercept	0.35	0.22	0.13	1.42	(0.91, 2.19)
Gender: Female					
Gender: Male	− 0.005	0.10	0.96	1.00	(0.82, 1.21)
Gender: Non-binary/third gender	− 0.01	0.14	0.93	0.99	(0.75, 1.31)
Age: < 25					
Age: 25–39	− 0.02	0.09	0.79	0.98	(0.81, 1.67)
Age: 40–55	− 0.03	0.17	0.87	0.97	(0.70, 1.34)
Age: > 55	0.33	0.17	0.06	1.39	(1.00, 1.93)
Employment: < 1 year					
Employment: 1–2 years	− 0.03	0.12	0.81	0.97	(0.77, 1.22)
Employment: > 2 years	− 0.16	0.11	0.17	0.85	(0.68, 1.10)
Repellent use: Never					
Repellent use: Sometimes	0.03	0.17	0.87	1.03	(0.74, 1.44)
Repellent use: Always	0.13	0.17	0.44	1.14	(0.82, 1.60)
Ticks on person: No					
Ticks on person: Yes	0.11	0.10	0.30	1.11	(0.91, 1.36)
Tick check: Never					
Tick check: Some days	0.06	0.13	0.66	1.06	(0.82, 1.36)
Tick check: Multiple times per day	− 0.24	0.14	0.11	0.79	(0.60, 1.05)
Tick check: Every day	0.01	0.11	0.89	1.01	(0.82, 1.26)
TBD concern: Not at all					
TBD concern: Somewhat	− 0.04	0.10	0.68	0.96	(0.80, 1.16)
TBD concern: Moderately	0.02	0.13	0.86	1.02	(0.80, 1.31)
TBD concern: Extremely	0.06	0.15	0.68	1.06	(0.80, 1.42)

Models were fit to individual infected tick encounter probabilities over both years across all sites, including all covariates that had variance inflation factors less than four as predictors. Model coefficients were exponentiated to obtain odds ratios and 95% confidence intervals.

**Table 5:** Full model results for generalized linear regression models evaluating the association between an individual’s predicted probability of encountering *Anaplasma phagocytophilum*-infected ticks and their survey responses averaged across all sampling sites.

	Estimate	SE	*p*-value	Odds ratio	95% CI
*All sites*					
Intercept	0.17	0.13	0.21	1.18	(0.92, 1.52)
Gender: Female					
Gender: Male	− 0.02	0.06	0.80	0.98	(0.88, 1.10)
Gender: Non-binary/third gender	− 0.01	0.08	0.95	0.99	(0.85, 1.17)
Age: < 25					
Age: 25–39	0.01	0.05	0.87	1.01	(0.91, 1.12)
Age: 40–55	0.02	0.10	0.82	1.02	(0.85, 1.23)
Age: > 55	0.27	0.10	0.01	1.32	(1.10, 1.59)
Employment: < 1 year					
Employment: 1–2 years	0.01	0.07	0.90	1.01	(0.88, 1.15)
Employment: > 2 years	− 0.08	0.07	0.24	0.92	(0.81, 1.05)
Repellent use: Never					
Repellent use: Sometimes	− 0.03	0.10	0.78	0.97	(0.80, 1.18)
Repellent use: Always	0.05	0.10	0.60	1.05	(0.87, 1.28)
Ticks on person: No					
Ticks on person: Yes	0.06	0.06	0.30	1.06	(0.95, 1.19)
Tick check: Never					
Tick check: Some days	− 0.02	0.07	0.75	0.98	(0.84, 1.13)
Tick check: Multiple times per day	− 0.11	0.08	0.19	0.89	(0.76, 1.05)
Tick check: Every day	− 0.01	0.06	0.85	0.99	(0.87, 1.12)
TBD concern: Not at all					
TBD concern: Somewhat	− 0.03	0.06	0.59	0.97	(0.87, 1.08)
TBD concern: Moderately	− 0.03	0.07	0.68	0.97	(0.84, 1.12)
TBD concern: Extremely	− 0.03	0.08	0.76	0.97	(0.83, 1.15)

Models were fit to individual infected tick encounter probabilities over both years across all sites, including all covariates that had variance inflation factors less than four as predictors. Model coefficients were exponentiated to obtain odds ratios and 95% confidence intervals.

## Discussion

Our study presents an approach to estimating the number of infected tick encounters among outdoor workers during their weekly job responsibilities, extending a novel method developed by Hassett et al. (2022). Our findings suggest that *B. burgdorferi* infection prevalence was extremely high in Lake Elmo Park Reserve, exceeding 60% in adults, and note that outdoor workers have a substantial probability of encountering infected ticks during their weekly job responsibilities. Previous research estimating tick-borne disease risks in human populations has relied on the entomological hazard index, which does not account for variation in human behavior that likely mediates the risk of infection (McClure and Diuk-Wasser, 2018). Here, we integrated active tick surveillance with self-reported worker behavior to assess individual-level exposure to infected ticks among occupationally exposed populations in Minnesota.

Total tick counts were highest in Lake Elmo Park Reserve, followed by Carlos Avery WMA and Whitewater WMA, consistent with previous reports of more established tick populations in northeastern Minnesota (Johnson et al., 2018a). However, when standardized by sampling effort, tick densities were highest in Carlos Avery WMA, followed by Lake Elmo Park Reserve and Whitewater WMA. Standardizing by sampling effort allowed for a refined characterization of tick population dynamics, demonstrating that raw tick counts alone may overestimate tick distributions. Blacklegged tick populations are known to be spatially heterogeneous in Minnesota, shaped by both abiotic and biotic factors (Cassens et al., 2023), with our collections emphasizing the interannual variability in tick abundance. Whether this variability is driven by environmental conditions, reservoir host dynamics, tick life history traits (e.g., diapause), or other factors remains uncertain. Future research should prioritize multifaceted sampling strategies, including collecting questing ticks, removing ticks from trapped animals, and passively collecting ticks encountered by outdoor workers. These collections could be coupled with environmental characteristics to provide a more explanatory picture of the factors influencing tick abundance, density, and infection prevalence in Minnesota.

In the Upper Midwest, the prevalence of tick-borne pathogens is consistently lower than in the Northeast, yet both represent primary foci of tick-borne disease incidence in the USA (Eisen and Eisen, 2018). In Minnesota, *B. burgdorferi* prevalence typically ranges from 17 to 25% in nymphs and 32–45% in adults, while *A. phagocytophilum* prevalence ranges from 6 to 10% in nymphs and 4–12% in adults (Foster et al., 2022; Johnson et al., 2018b). Our study found substantially elevated *B. burgdorferi* infection prevalence across all sites, averaging 36.5% in nymphs and 62.1% in adults. Conversely, *A. phagocytophilum* prevalence is closely aligned with previous estimates, averaging 7.1% in nymphs and 12.2% in adults. As expected, infection prevalence was higher in adult ticks than in nymphs due to the additional blood meal required for development, which increases their likelihood of acquiring pathogens. Notably, the infection prevalence in Lake Elmo Park Reserve was particularly high in 2023, when sample sizes were sufficient to reliably estimate pathogen levels. Given Lake Elmo Park Reserve’s proximity to western Wisconsin, where blacklegged ticks are thought to have been introduced and established in Minnesota by the 1980s (Dennis et al., 1998; Schrock, 1982), this region likely harbors some of the state’s longest-standing established tick populations, allowing enzootic pathogen transmission cycles to stabilize over time. While tick-borne pathogen transmission is inherently stochastic, further investigation is warranted to understand the abiotic (e.g., climate) and biotic (e.g., reservoir host composition) factors contributing to the elevated pathogen prevalence observed in this region.

By integrating active tick surveillance with outdoor workers’ self-reported exposure to tick habitats, we estimated the predicted probability of encountering infected ticks during weekly job responsibilities. This method assumes a passive sampling framework, where an individual’s risk is a function of their time spent in tick habitat and the density of infected ticks within those habitats. However, this measure does not account for variability in worker behavior across sites, which may limit generalizability. Despite this limitation, our study provides a foundation for assessing the occupational risks of infected tick encounters, aiding future research on risk factors and prevention strategies. Among the study sites, Carlos Avery WMA had the highest estimated weekly probability of infected tick encounters, followed by Lake Elmo Park Reserve and Whitewater WMA. Assuming constant worker behavior, the probability of encountering a *B. burgdorferi*-infected tick ranged from 25 to 56% across sites, averaging 38% (IQR 0.22–0.49) in all sites. This estimate is intrinsically influenced by the density of infected ticks, a product of infection prevalence and tick density. Considering the complexity of factors affecting tick abundance and pathogen prevalence, future research should directly track worker movements in tick habitats alongside long-term surveillance to better capture tick and pathogen population dynamics. Additionally, longitudinal seroprevalence studies in outdoor workers could provide insight into the relationship between predicted infected tick encounters and actual pathogen exposure.

Surveying outdoor workers through coupled active surveillance revealed how demographic and behavioral characteristics mediate infected tick encounters. For instance, female outdoor workers under 25 had the highest weekly probability of encountering infected ticks, which contrasts with the general population, where males demonstrate greater risk (Mead et al., 2024). This suggests that female outdoor workers may possess distinct risk profiles compared to the general population, necessitating further research. Additionally, our results show that workers above the age of 55 faced elevated risks compared to other age groups, consistent with a higher tick-borne disease incidence among this age group (Mead et al., 2024). Yet, our sample population contained only three individuals above the age of 55, limiting the generalizability of these findings and emphasizing the need for further investigation. Workers reporting greater concern about tick-borne diseases and frequent repellent use paradoxically had higher odds of encountering infected ticks, likely reflecting a correlation between self-reported exposure and perceived risk. Similarly, workers conducting frequent tick checks were more likely to encounter infected ticks, except those performing multiple tick checks per day. This ultimately stems from smaller sample sizes, where seven individuals reported multiple daily checks, all classified as low exposure, collectively reducing their predicted encounter probability. This suggests that workers who are more vigilant about tick checks may also engage in other protective behaviors and/or avoid tick habitats that reduce their overall exposure risk. However, associations between individual infected tick encounter probabilities and KAP responses did not meet conventional thresholds for statistical significance, requiring future research to validate our findings.

Our findings provide valuable insight into tick-borne disease risks among outdoor workers in Minnesota, however, several limitations may affect our analysis and interpretations. First, ticks were collected from single sampling sites within each location, which may not capture local heterogeneity in tick densities and infection prevalence. This spatial mismatch provides only a snapshot of potential tick encounters across the landscape. Further, our PCR assay to assess infection prevalence with *A. phagocytophilum* could not differentiate the human-active variant, leading to overestimates of human anaplasmosis disease risk from infected tick encounter probabilities. Second, without direct measurement of worker behavior in tick habitat, we assumed constant worker behavior across individuals and sites, potentially leading to exposure misclassification. Site-specific and individual differences in work activities influence tick exposure in ways not fully captured here. Third, reliance on self-reported exposure data introduces information bias through recall errors and variability in risk perceptions, affecting our estimates of infected tick encounter probability. Finally, the low survey responses rate and subsequent small sample size limited statistical power to detect subtle associations, particularly for subgroup analyses or interaction effects that may be important for understanding differential risk patterns across worker populations. Additionally, survey participation may be subject to selection bias if respondents had heightened awareness of tick-borne disease risks. Future studies incorporating direct behavioral observation, comprehensive spatial sampling, and larger representative samples would strengthen characterization of occupational tick exposure risks. There remains an urgent need to develop objective measurements capturing infected tick encounters among highly exposed populations.

## Conclusions

Using integrated tick surveillance and self-reported exposure data, our results provide the first occupational risk assessment of infected tick encounters among outdoor workers. Our study revealed a concerningly high prevalence of *B. burgdorferi* infection in Lake Elmo Park Reserve (49%), necessitating continued surveillance in this region. We identified substantial variation in infected tick encounter probabilities by site, with Carlos Avery WMA posing the highest weekly risk, followed by Lake Elmo Park Reserve and Whitewater WMA. Demographic factors were associated with the probability of encountering infected ticks, with individuals above 55 years of age having the highest odds of encountering infected ticks as part of their job responsibilities. Future studies should refine these estimates by incorporating direct worker movement tracking and longitudinal pathogen exposure assessments to enhance our understanding of occupational tick-borne disease risks.

## Supplementary Information

Below is the link to the electronic supplementary material.

Supplementary file 1 (PDF 61 kb) Questionnaire administered to outdoor workers.

Supplementary file2 (PDF 458 kb) Descriptive results for all ordinal regression analyses and mixed effects not reported in the manuscript

Supplementary file3 (PDF 396 kb) R code used for data analysis.
