# Supplementary material for: Estimating Infected Blacklegged Tick Encounters Among Outdoor Workers in Minnesota
Source: Ecohealth. 2025 Sep 18;23(1):137–52. doi: 10.1007/s10393-025-01753-7 (PMC12932337; doi:10.1007/s10393-025-01753-7)
Supplement: Supplementary file 1 — (PDF 61 kb) Questionnaire administered to outdoor workers. [file 10393_2025_1753_MOESM1_ESM.pdf]

MCOHS-ERC: Questionnaire

Participation in this study is voluntary. Your decision whether or not to participate will not affect your current or future relations with the University of Minnesota or your employer. If you decide to participate, you are free not to answer any question or withdraw at any time without affecting those relationships. Any personal information that could identify you will be removed or changed before we publish any report or share the results from this study. Your answers from the survey will be aggregated to ensure participants cannot be identified. All survey responses will be maintained by the researcher and will not be shared with other researchers. By checking this box, you are consenting to participate.

	Item	Responses
1.	Which gender do you identify with?	A. Female<br>B. Male<br>C. Non-binary / third gender<br>D. Prefer not to say
2.	What is your age?	A. < 25<br>B. 25-39<br>C. 40-55<br>D. > 55<br>E. Prefer not to say
3.	How long have you been employed at your current role?	A. < 1 year<br>B. 1-2 years<br>C. > 2 years
4.	During the months May-July, approximately how many hours per week do you spend in forested, edge, or trail habitats as part of your job responsibilities?	__________
5.	During the months May-July, approximately how many hours do you work per week?	__________
6.	When performing your outdoor job responsibilities, how often do you apply insect repellant or wear clothing that protects against mosquitos or ticks (e.g., permethrin-treated clothes)?	A. Always<br>B. Sometimes<br>C. Never

7.	When performing your outdoor job responsibilities, how often do you find ticks on yourself? (Either attached to your skin, your clothing, or freely moving)	A. > 2 times per week<br>B. 1-2 times per week<br>C. 0 times per week
8.	During or after performing outdoor job responsibilities, how often do you perform a tick check? (A tick check consists of examining your body for any attached or freely moving ticks)	A. Every day<br>B. Some days<br>C. Never
9.	When performing your outdoor job responsibilities, how concerned are you about getting a tick-borne disease?	A. Extremely concerned<br>B. Moderately concerned<br>C. Somewhat concerned<br>D. Not at all concerned
10.	Have you ever been diagnosed with a tick-borne disease (e.g., Lyme, anaplasmosis, babesiosis, etc.)?	A. No<br>B. Yes
11.	(If yes to 10) Were you diagnosed with a tick-borne disease in 2024?	A. No<br>B. Yes
12.	(If yes to 10) What tick-borne disease were you diagnosed with?	A. Lyme<br>B. Anaplasmosis<br>C. Babesiosis<br>D. Other<br>E. Do not know
