# Supplementary material for: Estimating Infected Blacklegged Tick Encounters Among Outdoor Workers in Minnesota
Source: Ecohealth. 2025 Sep 18;23(1):137–52. doi: 10.1007/s10393-025-01753-7 (PMC12932337; doi:10.1007/s10393-025-01753-7)
Supplement: Supplementary file 2 — Supplementary file2 (PDF 458 kb) Descriptive results for all ordinal regression analyses and mixed effects not reported in the manuscript [file 10393_2025_1753_MOESM2_ESM.pdf]

Supplementary Table S1. Ordinal regression model results for estimating the association between dichotomized self-reported exposure and survey responses, adjusted for age and gender. Model coefficients were exponentiated to provide odds ratios and their respective 95% confidence intervals.

	Estimate	SE	*p*-value	Odds ratio	95% CI
**Outcome: Repellant usage**					
High exposure to tick habitat	1.26	0.71	0.08	3.51	(0.90, 15.1)
Age: 25-39	-0.30	0.67	0.66	0.74	(0.19, 2.76)
Age: 40-55	1.04	1.30	0.42	2.84	(0.22, 40.0)
Age: >55	0.11	1.32	0.93	1.12	(0.08, 16.7)
Gender: Male	-1.41	0.73	0.05	0.25	(0.05, 0.97)
Gender: Non-binary / third gender	0.69	1.04	0.51	2.00	(0.27, 18.5)
**Outcome: Frequency of ticks found on oneself**					
High exposure to tick habitat	-0.94	0.71	0.18	0.39	(0.09, 1.53)
Age: 25-39	0.58	0.70	0.41	1.79	(0.46, 7.37)
Age: 40-55	1.67	1.16	0.15	5.33	(0.57, 56.7)
Age: >55	1.64	1.32	0.22	5.17	(0.41, 77.8)
Gender: Male	0.95	0.72	0.19	2.58	(0.65, 11.2)
Gender: Non-binary / third gender	1.07	1.07	0.32	2.92	(0.39, 27.4)
**Outcome: Frequency of tick checks performed**					
High exposure to tick habitat	0.56	0.70	0.42	1.75	(0.45, 7.36)
Age: 25-39	0.24	0.60	0.69	1.27	(0.40, 4.15)
Age: 40-55	-0.52	1.06	0.62	0.59	(0.07, 4.56)
Age: >55	0.43	1.39	0.76	1.54	(0.11, 41.1)
Gender: Male	-0.11	0.62	0.86	0.89	(0.27, 3.02)
Gender: Non-binary / third gender	0.62	0.98	0.53	1.85	(0.29, 15.7)
**Outcome: TBD concern**					
High exposure to tick habitat	0.56	0.65	0.38	1.76	(0.49, 6.44)
Age: 25-39	1.07	0.64	0.09	2.93	(0.85, 10.5)
Age: 40-55	0.83	1.09	0.45	2.29	(0.25, 19.4)
Age: >55	-0.69	1.16	0.55	0.50	(0.05, 4.9)
Gender: Male	0.02	0.64	0.98	1.02	(0.29, 3.57)
Gender: Non-binary / third gender	1.00	0.91	0.27	2.71	(0.47, 17.8)

**Supplementary Table S2. Full model results for generalized linear regression models evaluating the association between an individual's predicted probability of encountering *Borrelia burgdorferi*-infected ticks and their survey responses partitioned by sampling site. Models were fit to individual infected tick encounter probabilities over both years per site, including all covariates that had variance inflation factors less than four as predictors. Model coefficients were exponentiated to obtain odds ratios and 95% confidence intervals.**					
	**Estimate**	**SE**	***p*-value**	**Odds ratio**	**95% CI**
**Model 1: Washington County**					
Intercept	1.14	0.34	0.002	3.12	(1.60, 6.09)
Gender: Female	ref				
Gender: Male	-0.02	0.09	0.82	0.98	(0.82, 1.17)
Gender: Non-binary / third gender	-0.07	0.13	0.61	0.93	(0.72, 1.21)
Age: < 25	ref				
Age: 25-39	-0.08	0.09	0.40	0.93	(0.78, 1.10)
Age: 40-55	-0.14	0.16	0.37	0.87	(0.64, 1.18)
Age: >55	0.37	0.15	0.02	1.45	(1.07, 1.95)
Employment: < 1 year	ref				
Employment: 1-2 years	-0.12	0.11	0.30	0.89	(0.71, 1.11)
Employment: >2 years	-0.11	0.11	0.32	0.90	(0.73, 1.11)
Repellant use: Never	ref				
Repellant use: Sometimes	0.05	0.16	0.76	1.05	(0.77, 1.43)
Repellant use: Always	0.09	0.16	0.55	1.10	(0.81, 1.49)
Ticks on person: No	ref				
Ticks on person: Yes	0.03	0.10	0.73	1.03	(0.86, 1.25)
Tick check: Never	ref				
Tick check: Only in tick habitat	-0.87	0.30	0.01	0.42	(0.23, 0.75)
Tick check: Some days	-0.74	0.29	0.02	0.48	(0.27, 0.84)
Tick check: Multiple times per day	-1.02	0.30	0.002	0.36	(0.20, 0.65)
Tick check: Every day	-0.80	0.29	0.01	0.45	(0.25, 0.80)
TBD concern: Not at all	ref				
TBD concern: Somewhat	0.03	0.09	0.77	1.03	(0.56, 1.23)
TBD concern: Moderately	0.08	0.12	0.48	1.09	(0.86, 1.37)
TBD concern: Extremely	0.18	0.14	0.22	1.19	(0.91, 1.58)
**Model 2: Carlos Avery WMA**					
Intercept	1.20	0.37	0.003	3.30	(1.60, 6.84)
Gender: Female	ref				
Gender: Male	0.01	0.10	0.94	1.01	(0.83, 1.22)

Gender: Non-binary / third gender	-0.08	0.14	0.59	0.93	(0.70, 1.23)
Age: < 25	ref				
Age: 25-39	-0.12	0.09	0.20	0.88	(0.73, 1.06)
Age: 40-55	-0.22	0.17	0.21	0.80	(0.57, 1.12)
Age: >55	0.20	0.17	0.25	1.22	(0.88, 1.69)
Employment: < 1 year	ref				
Employment: 1-2 years	-0.17	0.12	0.19	0.85	(0.66, 1.01)
Employment: >2 years	-0.13	0.12	0.26	0.87	(0.70, 1.10)
Repellant use: Never	ref				
Repellant use: Sometimes	0.14	0.17	0.42	1.15	(0.82, 1.61)
Repellant use: Always	0.14	0.17	0.40	1.16	(0.83, 1.61)
Ticks on person: No	ref				
Ticks on person: Yes	0.04	0.11	0.71	1.04	(0.85, 1.28)
Tick check: Never	ref				
Tick check: Only in tick habitat	-0.82	0.32	0.02	0.44	(0.23, 0.82)
Tick check: Some days	-0.58	0.32	0.07	0.56	(0.30, 1.03)
Tick check: Multiple times per day	-1.02	0.32	0.004	0.36	(0.19, 0.68)
Tick check: Every day	-0.72	0.32	0.03	0.49	(0.26, 0.91)
TBD concern: Not at all	ref				
TBD concern: Somewhat	0.04	0.10	0.70	1.04	(0.86, 1.26)
TBD concern: Moderately	0.17	0.13	0.20	1.19	(0.92, 1.53)
TBD concern: Extremely	0.29	0.15	0.07	1.34	(1.00, 1.81)
**Model 3: Whitewater WMA**					
Intercept	1.03	0.31	0.002	2.81	(1.54, 5.14)
Gender: Female	ref				
Gender: Male	-0.03	0.08	0.72	0.97	(0.83, 1.14)
Gender: Non-binary / third gender	-0.06	0.12	0.59	0.94	(0.74, 1.18)
Age: < 25	ref				
Age: 25-39	-0.05	0.08	0.52	0.95	(0.81, 1.11)
Age: 40-55	-0.10	0.14	0.48	0.90	(0.68, 1.19)
Age: >55	0.40	0.14	0.01	1.50	(1.14, 1.97)
Employment: < 1 year	ref				
Employment: 1-2 years	-0.09	0.10	0.39	0.91	(0.75, 1.12)
Employment: >2 years	-0.08	0.10	0.39	0.92	(0.76, 1.11)
Repellant use: Never	ref				
Repellant use: Sometimes	0.01	0.14	0.96	1.01	(0.76, 1.33)
Repellant use: Always	0.06	0.14	0.65	1.07	(0.81. 1.41)

Ticks on person: No	ref				
Ticks on person: Yes	0.03	0.09	0.75	1.03	(0.87, 1.22)
Tick check: Never	ref				
Tick check: Only in tick habitat	-0.82	0.27	0.005	0.44	(0.26, 0.74)
Tick check: Some days	-0.75	0.26	0.01	0.47	(0.28, 0.79)
Tick check: Multiple times per day	-0.93	0.27	0.002	0.40	(0.23, 0.67)
Tick check: Every day	-0.77	0.26	0.01	0.46	(0.28, 0.78)
TBD concern: Not at all	ref				
TBD concern: Somewhat	0.02	0.08	0.80	1.02	(0.87, 1.20)
TBD concern: Moderately	0.04	0.11	0.70	1.04	(0.85, 1.29)
TBD concern: Extremely	0.11	0.13	0.39	1.12	(0.87, 1.44)

**Supplementary Table S3. Generalized linear model results evaluating the association between individual predicted probabilities of encountering *Borrelia burgdorferi*-infected ticks and survey responses. Model selection was performed (i.e., stepwise selection) to identify predictive models that minimized AIC values. Model coefficients were exponentiated to obtain odds ratios and their 95% confidence intervals.**					
	**Estimate**	**SE**	***p*-value**	**Odds ratio**	**95% CI**
**Model 1: Washington County**					
Intercept	1.16	0.23	0.00001	3.18	(2.03, 4.98)
Age: < 25	ref				
Age: 25-39	-0.08	0.07	0.250	0.93	(0.81, 1.05)
Age: 40-55	-0.17	0.11	0.137	0.84	(0.67, 1.05)
Age: >55	0.31	0.12	0.016	1.37	(1.07, 1.74)
Tick check: Never	ref				
Tick check: Only in tick habitat	-0.84	0.23	0.001	0.43	(0.27, 0.68)
Tick check: Some days	-0.75	0.23	0.003	0.47	(0.30, 0.75)
Tick check: Multiple times per day	-0.94	0.23	0.0002	0.39	(0.25, 0.61)
Tick check: Every day	-0.77	0.23	0.002	0.46	(0.30, 0.73)
**Model 2: Carlos Avery WMA**					
Intercept	1.26	0.26	0.00002	3.51	(2.11, 5.86)
Age: < 25	ref				
Age: 25-39	-0.11	0.08	0.161	0.90	(0.77, 1.04)
Age: 40-55	-0.26	0.13	0.056	0.77	(0.60, 1.00)
Age: >55	0.12	0.14	0.398	1.13	(0.85, 1.49)
Tick check: Never	ref				
Tick check: Only in tick habitat	-0.74	0.27	0.008	0.48	(0.28, 0.80)
Tick check: Some days	-0.56	0.27	0.042	0.57	(0.34, 0.96)
Tick check: Multiple times per day	-0.87	0.26	0.002	0.42	(0.25, 0.69)
Tick check: Every day	-0.63	0.26	0.021	0.54	(0.32, 0.89)
**Model 3: Whitewater WMA**					
Intercept	1.03	0.20	0.00001	2.80	(1.88, 4.18)
Age: < 25	ref				
Age: 25-39	-0.06	0.06	0.315	0.94	(0.84, 1.06)
Age: 40-55	-0.13	0.10	0.223	0.88	(0.72, 1.08)

Age: >55	0.36	0.11	0.002	1.44	(1.16, 1.79)
Tick check: Never	ref				
Tick check: Only in tick habitat	-0.81	0.21	0.0004	0.44	(0.30, 0.67)
Tick check: Some days	-0.76	0.21	0.0008	0.47	(0.31, 0.70)
Tick check: Multiple times per day	-0.89	0.20	0.0001	0.41	(0.28, 0.61)
Tick check: Every day	-0.76	0.20	0.0006	0.47	(0.31, 0.70)

**Supplementary Table S4. Generalized linear model results evaluating the association between individual predicted probabilities of encountering Borrelia burgdorferi-infected ticks and survey responses. Model selection was performed using multi-model inference to identify predictive models that minimized AIC values. In multi-model inference, models with a change in AIC <4 are averaged to obtain coefficient estimates. Model coefficients represent full averages. Model coefficients were exponentiated to obtain odds ratios and their 95% confidence intervals.**					
	**Estimate**	**SE**	***p*-value**	**Odds ratio**	**95% CI**
**Model 1: Washington County**					
Intercept	1.13	0.24	0.000004	3.10	(1.92, 4.99)
Age: < 25	ref				
Age: 25-39	-0.07	0.07	0.34	0.94	(0.82, 1.07)
Age: 40-55	-0.15	0.12	0.24	0.86	(0.67, 1.10)
Age: >55	0.27	0.16	0.10	1.31	(0.95, 1.80)
Tick check: Never	ref				
Tick check: Only in tick habitat	-0.81	0.24	0.001	0.44	(0.27, 0.72)
Tick check: Some days	-0.73	0.24	0.003	0.48	(0.30, 0.78)
Tick check: Multiple times per day	-0.93	0.23	0.0001	0.39	(0.25, 0.63)
Tick check: Every day	-0.74	0.24	0.002	0.48	(0.30, 0.77)
Ticks on person: No	ref				
Ticks on person: Yes	0.00	0.03	0.99	1.00	(0.95, 1.05)
**Model 2: Carlos Avery WMA**					
Intercept	1.07	0.27	0.0001	2.90	(1.69, 4.98)
Age: < 25	ref				
Age: 25-39	-0.02	0.06	0.67	0.98	(0.87, 1.09)
Age: 40-55	-0.06	0.12	0.64	0.94	(0.74, 1.21)
Age: >55	0.03	0.08	0.75	1.03	(0.87, 1.22)
Tick check: Never	ref				
Tick check: Only in tick habitat	-0.57	0.27	0.04	0.57	(0.33, 0.98)
Tick check: Some days	-0.39	0.27	0.15	0.68	(0.40, 1.16)
Tick check: Multiple times per day	-0.76	0.26	0.005	0.47	(0.28, 0.80)
Tick check: Every day	-0.45	0.26	0.10	0.64	(0.37, 1.08)
Ticks on person: No	ref				

Ticks on person: Yes	0.004	0.03	0.91	1.00	(0.94, 1.07)
Employment: < 1 year	ref				
Employment: 1-2 years	-0.01	0.05	0.83	0.99	(0.90, 1.09)
Employment: > 2 years	-0.02	0.05	0.76	0.98	(0.89, 1.09)
**Model 3: Whitewater WMA**					
Intercept	1.03	0.21	0.000001	2.81	(1.85, 4.25)
Age: < 25	ref				
Age: 25-39	-0.06	0.06	0.33	0.94	(0.84, 1.06)
Age: 40-55	-0.13	0.10	0.24	0.88	(0.72, 1.09)
Age: >55	0.37	0.11	0.001	1.44	(1.15, 1.81)
Tick check: Never	ref				
Tick check: Only in tick habitat	-0.81	0.21	0.0002	0.44	(0.29, 0.68)
Tick check: Some days	-0.76	0.21	0.0004	0.47	(0.31, 0.71)
Tick check: Multiple times per day	-0.89	0.20	0.00002	0.41	(0.27, 0.62)
Tick check: Every day	-0.76	0.20	0.0003	0.47	(0.31, 0.70)
Ticks on person: No	ref				
Tick on person: Yes	0.00	0.03	0.90	1.00	(0.94, 1.05)

**Supplementary Table S5. Estimated marginal means for the predicted probability of encountering *Borrelia burgdorferi*-infected ticks for each level of predictor in the generalized linear models using stepwise selection in Table S3.**						
	**Washington County**		**Carlos Avery WMA**		**Whitewater WMA**	
	**Predicted**	**95% CI**	**Predicted**	**95% CI**	**Predicted**	**95% CI**
**Age: < 25**						
Frequency of tick check						
Never	1.16	(0.69, 1.62)	1.26	(0.73, 1.78)	1.03	(0.62, 1.44)
Only when I am in tick habitat	0.31	(0.15, 0.48)	0.52	(0.33, 0.70)	0.22	(0.07, 0.36)
Some days	0.41	(0.29, 0.53)	0.7	(0.56, 0.84)	0.27	(0.16, 0.38)
Multiple times a day	0.21	(0.03, 0.39)	0.38	(0.18, 0.59)	0.14	(-0.02, 0.30)
Every day	0.39	(0.28, 0.50)	0.63	(0.51, 0.76)	0.27	(0.17, 0.36)
**Age: 25-39**						
Frequency of tick check						
Never	1.08	(0.61, 1.54)	1.15	(0.62, 1.68)	0.97	(0.56, 1.38)
Only when I am in tick habitat	0.24	(0.05, 0.43)	0.41	(0.20, 0.63)	0.16	(-0.01, 0.33)
Some days	0.33	(0.19, 0.47)	0.59	(0.43, 0.75)	0.21	(0.09, 0.33)
Multiple times a day	0.13	(-0.04, 0.31)	0.28	(0.08, 0.48)	0.08	(-0.08, 0.24)
Every day	0.31	(0.18, 0.44)	0.52	(0.38, 0.67)	0.21	(0.09, 0.32)
**Age: 40-55**						
Frequency of tick check						
Never	0.98	(0.58, 1.38)	1	(0.54, 1.46)	0.9	(0.55, 1.26)
Only when I am in	0.14	(-0.11, 0.39)	0.26	(-0.02, 0.54)	0.09	(-0.13, 0.31)

tick habitat						
Some days	0.24	(-0.02, 0.49)	0.44	(0.16, 0.73)	0.14	(-0.08, 0.37)
Multiple times a day	0.04	(-0.18, 0.26)	0.13	(-0.12, 0.38)	0.01	(-0.18, 0.21)
Every day	0.21	(-0.02, 0.45)	0.37	(0.11, 0.64)	0.14	(-0.07, 0.35)
**Age: > 55**						
Frequency of tick check						
Never	1.47	(0.95, 1.98)	1.38	(0.79, 1.96)	1.39	(0.94, 1.85)
Only when I am in tick habitat	0.63	(0.37, 0.89)	0.64	(0.34, 0.94)	0.58	(0.35, 0.82)
Some days	0.72	(0.45, 0.99)	0.82	(0.51, 1.13)	0.63	(0.39, 0.88)
Multiple times a day	0.52	(0.23, 0.82)	0.51	(0.17, 0.84)	0.5	(0.24, 0.77)
Every day	0.7	(0.46, 0.94)	0.75	(0.48, 1.02)	0.63	(0.42, 0.84)

**Supplementary Table S6. Full model results for generalized linear regression models evaluating the association between an individual's predicted probability of encountering *Anaplasma phagocytophilum*-infected ticks and their survey responses partitioned by sampling site. Models were fit to individual infected tick encounter probabilities over both years per site, including all covariates that had variance inflation factors less than four as predictors. Model coefficients were exponentiated to obtain odds ratios and 95% confidence intervals.**					
	**Estimate**	**SE**	***p*-value**	**Odds ratio**	**95% CI**
**Model 1: Washington County**					
Intercept	0.61	0.18	0.002	1.84	(1.29, 2.65)
Gender: Male	-0.02	0.05	0.64	0.98	(0.89, 1.08)
Gender: Non-binary / third gender	-0.04	0.07	0.57	0.96	(0.84, 1.10)
Age: < 25	ref				
Age: 25-39	-0.02	0.05	0.67	0.98	(0.89, 1.07)
Age: 40-55	-0.05	0.09	0.60	0.96	(0.81, 1.13)
Age: >55	0.28	0.08	0.00	1.32	(1.12, 1.55)
Employment: < 1 year	ref				
Employment: 1-2 years	-0.04	0.06	0.50	0.96	(0.85, 1.08)
Emplyment: >2 years	-0.04	0.06	0.48	0.96	(0.86, 1.07)
Repellant use: Never	ref				
Repellant use: Sometimes	-0.02	0.08	0.85	0.98	(0.83, 1.16)
Repellant use: Always	0.03	0.08	0.76	1.03	(0.87, 1.21)
Ticks on person: No	ref				
Ticks on person: Yes	0.02	0.05	0.77	1.02	(0.92, 1.12)
Tick check: Never	ref				
Tick check: Only in tick habitat	-0.50	0.16	0.004	0.61	(0.44, 0.83)
Tick check: Some days	-0.48	0.16	0.004	0.62	(0.45, 0.84)
Tick check: Multiple times per day	-0.55	0.16	0.002	0.58	(0.42, 0.79)
Tick check: Every day	-0.48	0.16	0.01	0.62	(0.45, 0.84)
TBD concern: Not at all	ref				
TBD concern: Somewhat	0.01	0.05	0.84	1.01	(0.92, 1.11)
TBD concern: Moderately	0.00	0.06	0.94	1.01	(0.89, 1.14)
TBD concern: Extremely	0.04	0.08	0.63	1.04	(0.89, 1.21)
**Model 2: Carlos Avery WMA**					
Intercept	1.05	0.31	0.002	2.85	(1.55, 5.25)
Gender: Male	-0.03	0.08	0.73	0.97	(0.82, 1.14)
Gender: Non-binary / third gender	-0.06	0.12	0.60	0.94	(0.74, 1.19)

Age: < 25	ref				
Age: 25-39	-0.05	0.08	0.51	0.95	(0.81, 1.11)
Age: 40-55	-0.11	0.14	0.47	0.90	(0.68, 1.19)
Age: >55	0.40	0.14	0.01	1.50	(1.14, 1.97)
Employment: < 1 year	ref				
Employment: 1-2 years	-0.09	0.10	0.38	0.91	(0.74, 1.12)
Emplyment: >2 years	-0.09	0.10	0.38	0.92	(0.76, 1.11)
Repellant use: Never	ref				
Repellant use: Sometimes	0.01	0.14	0.94	1.01	(0.76, 1.34)
Repellant use: Always	0.07	0.14	0.65	1.07	(0.81, 1.42)
Ticks on person: No	ref				
Ticks on person: Yes	0.03	0.09	0.75	1.03	(0.67, 1.22)
Tick check: Never	ref				
Tick check: Only in tick habitat	-0.83	0.27	0.005	0.44	(0.26, 0.74)
Tick check: Some days	-0.75	0.26	0.01	0.47	(0.28, 0.79)
Tick check: Multiple times per day	-0.94	0.27	0.002	0.39	(0.23, 0.66)
Tick check: Every day	-0.78	0.27	0.01	0.46	(0.27, 0.78)
TBD concern: Not at all	ref				
TBD concern: Somewhat	0.02	0.08	0.80	1.02	(0.87, 1.20)
TBD concern: Moderately	0.05	0.11	0.68	1.05	(0.85, 1.29)
TBD concern: Extremely	0.12	0.13	0.37	1.12	(0.87, 1.45)
**Model 3: Whitewater WMA**					
Intercept	0.26	0.08	0.003	1.30	(1.11, 1.52)
Gender: Male	-0.01	0.02	0.61	0.99	(0.95, 1.03)
Gender: Non-binary / third gender	-0.02	0.03	0.57	0.98	(0.92, 1.04)
Age: < 25	ref				
Age: 25-39	-0.01	0.02	0.71	0.99	(0.95, 1.03)
Age: 40-55	-0.02	0.04	0.64	0.98	(0.91, 1.06)
Age: >55	0.12	0.04	0.002	1.13	(1.06, 1.22)
Employment: < 1 year	ref				
Employment: 1-2 years	-0.02	0.03	0.54	0.98	(0.93, 1.04)
Emplyment: >2 years	-0.02	0.03	0.51	0.98	(0.94, 1.03)
Repellant use: Never	ref				
Repellant use: Sometimes	-0.01	0.04	0.79	0.99	(0.92, 1.06)
Repellant use: Always	0.01	0.04	0.80	1.01	(0.94, 1.08)
Ticks on person: No	ref				
Ticks on person: Yes	0.01	0.02	0.77	1.01	(0.96, 1.05)
Tick check: Never	ref				

Tick check: Only in tick habitat	-0.22	0.07	0.004	0.81	(0.70, 0.92)
Tick check: Some days	-0.21	0.07	0.004	0.81	(0.71, 0.92)
Tick check: Multiple times per day	-0.24	0.07	0.002	0.79	(0.69, 0.90)
Tick check: Every day	-0.21	0.07	0.005	0.81	(0.71, 0.93)
TBD concern: Not at all	ref				
TBD concern: Somewhat	0.004	0.02	0.85	1.00	(0.96, 1.05)
TBD concern: Moderately	-0.0004	0.03	0.99	1.00	(0.95, 1.06)
TBD concern: Extremely	0.01	0.03	0.72	1.01	(0.95, 1.08)

**Supplementary Table S7. Generalized linear model results evaluating the association between individual predicted probabilities of encountering *Anaplasma phagocytophilum*-infected ticks and survey responses. Model selection was performed (i.e., stepwise selection) to identify predictive models that minimized AIC values. Model coeffecients were exponentiated to obtain odds ratios and their 95% confidence intervals.**					
	**Estimate**	**SE**	***p*-value**	**Odds ratio**	**95% CI**
**Model 1: Washington County**					
Intercept	0.60	0.12	0.00002	1.82	(1.43, 2.31)
Age: < 25	ref				
Age: 25-39	-0.03	0.04	0.401	0.97	(0.91, 1.04)
Age: 40-55	-0.06	0.06	0.357	0.95	(0.84, 1.06)
Age: >55	0.26	0.07	0.0003	1.30	(1.14, 1.48)
Tick check: Never	ref				
Tick check: Only in tick habitat	-0.50	0.12	0.0002	0.60	(0.48, 0.77)
Tick check: Some days	-0.50	0.12	0.0003	0.61	(0.48, 0.78)
Tick check: Multiple times per day	-0.54	0.12	0.0001	0.58	(0.46, 0.74)
Tick check: Every day	-0.49	0.12	0.0002	0.61	(0.48, 0.78)
**Model 2: Carlos Avery WMA**					
Intercept	1.04	0.21	0.00001	2.84	(1.90, 4.26)
Age: < 25	ref				
Age: 25-39	-0.06	0.06	0.310	0.94	(0.84, 1.06)
Age: 40-55	-0.13	0.10	0.215	0.88	(0.72, 1.07)
Age: >55	0.36	0.11	0.002	1.44	(1.15, 1.79)
Tick check: Never	ref				
Tick check: Only in tick habitat	-0.82	0.21	0.0004	0.44	(0.29, 0.67)
Tick check: Some days	-0.76	0.21	0.001	0.47	(0.31, 0.70)
Tick check: Multiple times per day	-0.90	0.20	0.0001	0.41	(0.27, 0.61)
Tick check: Every day	-0.77	0.21	0.0006	0.46	(0.31, 0.70)
**Model 3: Whitewater WMA**					
Intercept	0.26	0.05	0.00002	1.29	(1.17, 1.43)
Age: < 25	ref				
Age: 25-39	-0.01	0.02	0.431	0.99	(0.96, 1.02)

Age: 40-55	-0.02	0.03	0.408	0.98	(0.93, 1.03)
Age: >55	0.12	0.03	0.0002	1.13	(1.07, 1.19)
Tick check: Never	ref				
Tick check: Only in tick habitat	-0.22	0.05	0.0002	0.80	(0.72, 0.90)
Tick check: Some days	-0.22	0.05	0.0002	0.80	(0.72, 0.89)
Tick check: Multiple times per day	-0.24	0.05	0.0001	0.79	(0.71, 0.87)
Tick check: Every day	-0.22	0.05	0.0002	0.81	(0.73, 0.89)

**Supplementary Table S8. Generalized linear model results evaluating the association between individual predicted probabilities of encountering *Anaplasma phagocytophilum*-infected ticks and survey responses. Model selection was performed using multi-model inference to identify predictive models that minimized AIC values. In multi-model inference, models with a change in AIC <4 are averaged to obtain coefficient estimates. Model coefficients represent full averages. Model coefficients were exponentiated to obtain odds ratios and their 95% confidence intervals.**					
	**Estimate**	**SE**	***p*-value**	**Odds ratio**	**95% CI**
**Model 1: Washington County**					
Intercept	0.60	0.13	0.000004	1.82	(1.41, 2.35)
Age: < 25	ref				
Age: 25-39	-0.03	0.04	0.42	0.97	(0.90, 1.04)
Age: 40-55	-0.06	0.06	0.37	0.94	(0.83, 1.07)
Age: >55	0.26	0.07	0.0001	1.30	(1.14, 1.49)
Tick check: Never	ref				
Tick check: Only in tick habitat	-0.50	0.12	0.0001	0.60	(0.47, 0.78)
Tick check: Some days	-0.49	0.12	0.0001	0.61	(0.47, 0.78)
Tick check: Multiple times per day	-0.54	0.12	0.00001	0.58	(0.46, 0.74)
Tick check: Every day	-0.49	0.12	0.0001	0.61	(0.48, 0.79)
Ticks on person: No	ref				
Ticks on person: Yes	-0.004	0.02	0.84	1.00	(0.96, 1.03)
Repellant use: Never	ref				
Repellant use: Sometimes	-0.002	0.02	0.93	1.00	(0.95, 1.04)
Repellant use: Always	0.004	0.02	0.88	1.00	(0.96, 1.05)
**Model 2: Carlos Avery WMA**					
Intercept	1.05	0.21	0.000001	2.85	(1.87, 4.34)
Age: < 25	ref				
Age: 25-39	-0.06	0.06	0.32	0.94	(0.83, 1.06)
Age: 40-55	-0.13	0.10	0.23	0.88	(0.71, 1.08)
Age: >55	0.36	0.11	0.002	1.44	(1.15, 1.81)
Tick check: Never	ref				
Tick check: Only in tick habitat	-0.82	0.21	0.0002	0.44	(0.29, 0.68)
Tick check: Some days	-0.76	0.21	0.0005	0.47	(0.30, 0.71)

Tick check: Multiple times per day	-0.90	0.20	0.00002	0.41	(0.27, 0.62)
Tick check: Every day	-0.77	0.21	0.0003	0.46	(0.31, 0.70)
Ticks on person: No	ref				
Ticks on person: Yes	0.00	0.03	0.91	1.00	(0.94, 1.05)
**Model 3: Whitewater WMA**					
Intercept	0.26	0.05	0.000005	1.29	(1.16, 1.44)
Age: < 25	ref				
Age: 25-39	-0.01	0.02	0.45	0.99	(0.96, 1.02)
Age: 40-55	-0.02	0.03	0.42	0.98	(0.93, 1.03)
Age: >55	0.12	0.03	0.0001	1.13	(1.06, 1.19)
Tick check: Never	ref				
Tick check: Only in tick habitat	-0.22	0.05	0.0001	0.80	(0.72, 0.89)
Tick check: Some days	-0.22	0.05	0.0001	0.80	(0.72, 0.90)
Tick check: Multiple times per day	-0.23	0.05	0.00001	0.79	(0.71, 0.88)
Tick check: Every day	-0.21	0.05	0.0001	0.81	(0.72, 0.90)
Ticks on person: No	ref				
Ticks on person: Yes	-0.002	0.01	0.83	1.00	(0.98, 1.01)
Repellant use: Never	ref				
Repellant use: Sometimes	-0.001	0.01	0.92	1.00	(0.98, 1.02)
Repellant use: Always	0.002	0.01	0.88	1.00	(0.98, 1.02)

**Supplementary Table S9. Estimated marginal means for the predicted probability of encountering *Anaplasma phagocytophilum*-infected ticks for each level of predictor in the generalized linear models using stepwise selection in Table S7.**						
	**Washington County**		**Carlos Avery WMA**		**Whitewater WMA**	
	**Predicted**	**95% CI**	**Predicted**	**95% CI**	**Predicted**	**95% CI**
**Age: < 25**						
Frequency of tick check						
Never	0.60	(0.35, 0.84)	1.04	(0.63, 1.46)	0.26	(0.15, 0.36)
Only when I am in tick habitat	0.09	(0.01, 0.18)	0.23	(0.08, 0.37)	0.04	(0.00, 0.07)
Some days	0.10	(0.04, 0.17)	0.28	(0.17, 0.39)	0.04	(0.01, 0.06)
Multiple times a day	0.06	(-0.04, 0.15)	0.15	(-0.02, 0.31)	0.02	(-0.02, 0.06)
Every day	0.11	(0.05, 0.17)	0.28	(0.18, 0.37)	0.04	(0.02, 0.07)
**Age: 25-39**						
Frequency of tick check						
Never	0.57	(0.32, 0.81)	0.98	(0.56, 1.40)	0.24	(0.14, 0.35)
Only when I am in tick habitat	0.06	(-0.04, 0.16)	0.17	(0.00, 0.34)	0.02	(-0.02, 0.07)
Some days	0.07	(0.00, 0.15)	0.22	(0.09, 0.34)	0.02	(-0.01, 0.06)
Multiple times a day	0.03	(-0.07, 0.12)	0.08	(-0.07, 0.24)	0.01	(-0.03, 0.05)
Every day	0.08	(0.01, 0.15)	0.21	(0.10, 0.33)	0.03	(0.00, 0.06)
**Age: 40-55**						
Frequency of tick check						
Never	0.54	(0.33, 0.75)	0.91	(0.55, 1.28)	0.23	(0.14, 0.33)
Only when I am in	0.04	(-0.09, 0.17)	0.10	(-0.13, 0.32)	0.01	(-0.04, 0.07)

tick habitat						
Some days	0.05	(-0.09, 0.18)	0.15	(-0.08, 0.38)	0.01	(-0.04, 0.07)
Multiple times a day	0.00	(-0.12, 0.12)	0.02	(-0.18, 0.21)	0.00	(-0.05, 0.05)
Every day	0.05	(-0.07, 0.18)	0.15	(-0.06, 0.35)	0.02	(-0.03, 0.07)
**Age: > 55**						
Frequency of tick check						
Never	0.86	(0.59, 1.13)	1.41	(0.94, 1.87)	0.38	(0.26, 0.49)
Only when I am in tick habitat	0.35	(0.22, 0.49)	0.59	(0.35, 0.83)	0.15	(0.09, 0.21)
Some days	0.36	(0.22, 0.51)	0.64	(0.40, 0.89)	0.16	(0.09, 0.22)
Multiple times a day	0.32	(0.16, 0.47)	0.51	(0.24, 0.77)	0.14	(0.07, 0.21)
Every day	0.37	(0.24, 0.50)	0.64	(0.42, 0.86)	0.16	(0.10, 0.21)
