# Supplementary material for: Estimating Infected Blacklegged Tick Encounters Among Outdoor Workers in Minnesota
Source: Ecohealth. 2025 Sep 18;23(1):137–52. doi: 10.1007/s10393-025-01753-7 (PMC12932337; doi:10.1007/s10393-025-01753-7)
Supplement: Supplementary file 3 — Supplementary file3 (PDF 396 kb) R code used for data analysis. [file 10393_2025_1753_MOESM3_ESM.pdf]

```
---
title: "Estimating infected blacklegged tick encounters among outdoor workers"
author: "Jacob Cassens"
date: "2025-08-07"
output: pdf_document
---


#### Generate table 1 summary statistics on survey responses
## read in survey responses spreadsheet
library(readxl)
surv<-
read_excel("/Users/jtcassens/Desktop/umn/research/dissertation/aim_1/survey_responses.xlsx",
sheet="clean_survey_responses")
View(surv)
str(surv)

## recoding categorical variables as factors
surv$Frequency_ticks_on_person <- factor(surv$Frequency_ticks_on_person, levels=c("0 times
per week", "1-2 times per week", "> 2 times per week"))
surv$Employment <- factor(surv$Employment, levels=c("< 1 year", "1-2 years", "> 2 years"))
surv$Repellant_use <- factor(surv$Repellant_use, levels=c("Never", "Sometimes", "Always"))
surv$Frequency_tick_check <- factor(surv$Frequency_tick_check, levels=c("Never", "Only
when I am in tick habitat", "Some days", "Multiple times a day", "Every day"))
surv$TBD_concern <- factor(surv$TBD_concern, levels=c("Not at all concerned", "Somewhat
concerned", "Moderately concerned", "Extremely concerned"))
surv$TBD_diagnosis_anytime <- factor(surv$TBD_diagnosis_anytime, levels=c("No", "Yes"))
surv$Age <- factor(surv$Age, levels=c("< 25", "25-39", "40-55", "> 55"))
surv$Gender <- factor(surv$Gender, levels=c("Female", "Male", "Non-binary / third
gender"))

### total table 1
library(tableone)
categorical_vars <- c("Gender", "Age", "Employment", "Repellant_use",
"Frequency_ticks_on_person", "Frequency_tick_check", "TBD_concern",
"TBD_diagnosis_anytime")
table1 <- CreateTableOne(vars=names(surv), data=surv, factorVars=categorical_vars)
print(table1, showAllLevels = TRUE)

## cut exposure fraction by median value (high vs low) for table 1
surv$Exposure_group_binary <- cut(surv$Exposure_fraction_computed,
                                  breaks=c(-Inf, median(surv$Exposure_fraction_computed),
Inf),
                                  labels=c("Low exposure to tick habitat", "High exposure
to tick habitat"))
View(surv)

table1_with_exposure_binary <- CreateTableOne(vars=names(surv)[!names(surv) %in%
c("Exposure_fraction_computed", "Exposure_group_binary")],
                                              strata="Exposure_group_binary",
                                              data=surv,
                                              factorVars=categorical_vars)
print(table1_with_exposure_binary, showAllLevels = TRUE, includeNA = TRUE)


#### creating figure 2 - NIP partitioned by site and year
### reshape dataset to long format for ease of creating figure
library(tidyr)
library(dplyr)
library(ggplot2)
library(lemon)
tick_long <- tick %>%
  pivot_longer(cols = c(nip_bb_23, aip_bb_23, tip_bb_23, nip_bb_24, aip_bb_24, tip_bb_24,
                        nip_bb_all, aip_bb_all, tip_bb_all, nip_ap_23, aip_ap_23,
```

```
tip_ap_23,
                        nip_ap_24, aip_ap_24, tip_ap_24, nip_ap_all, aip_ap_all,
tip_ap_all),
               names_to = "variable",
               values_to = "value") %>%
  separate(variable, into = c("type", "pathogen", "year"), sep = "_") %>%
  mutate(site = recode(site,
                       "wsh_ct" = "Lake Elmo Park Reserve",
                       "carlos_av" = "Carlos Avery WMA",
                       "whitewater" = "Whitewater WMA")) %>%
  mutate(year = recode(year,
                       "23" = "2023",
                       "24" = "2024",
                       "all" = "Total")) %>%
  mutate(pathogen = recode(pathogen,
                           "bb" = "Borrelia burgdorferi",
                           "ap" = "Anaplasma phagocytophilum"))
View(tick_long)
tick_long$type <- factor(tick_long$type, levels=c("nip", "aip", "tip"))
tick_long$site <- factor(tick_long$site, levels=c("Carlos Avery WMA", "Lake Elmo Park
Reserve", "Whitewater WMA"))

## create plot - wrapped by site
mcohs_figure2 <- ggplot(tick_long, aes(x = year, y = value, fill = type)) +
  geom_bar(stat = "identity", position = "dodge") +
  labs(x = "", y = "Infection prevalence (%)", fill = "Life stage") +
  scale_fill_manual(values = c("gray53", "indianred4", "darkgoldenrod2"),
                    labels = c("Nymph", "Adult", "Total")) +
  scale_y_continuous(labels = scales::percent_format(accuracy = 1),
                     expand = expansion(mult = c(0, 0.05))) +
  facet_grid(factor(pathogen, levels = c("Borrelia burgdorferi", "Anaplasma
phagocytophilum")) ~ site, scales = "free_y") + # Reorder facets
  theme_bw() +
  theme(axis.text.x = element_text(hjust = 0.5),
        axis.title.x = element_text(color = "black", size = 14),
        axis.title.y = element_text(color = "black", size = 14, face = "bold"),
        axis.text = element_text(color = "black", size = 14),
        strip.text.x = element_text(color = "black", size = 14, face = "bold"),
        strip.text.y = element_text(color = "black", size = 14, face = "bold.italic"),
        legend.title = element_text(color = "black", size = 14, face = "bold"),
        legend.text = element_text(color = "black", size = 12),
        panel.grid.major.x = element_blank(),
        panel.grid.minor.x = element_blank(),
        panel.grid.major.y = element_line(color = "gray90"),
        panel.grid.minor.y = element_line(color = "gray90"),
        panel.border = element_rect(color = "black", fill = NA, linewidth = 0.5),
        panel.spacing = unit(0.3, "lines"),
        panel.spacing.y = unit(1.5, "lines"),
        plot.background = element_rect(fill = 'white', color = NA))
mcohs_figure2
ggsave(filename = "mcohs_Figure2.png", plot = mcohs_figure2,  width = 9.5, height = 7, dpi
= 600)

#### glms to test stuff
View(surv)
test_age <- glm(Exposure_per_week ~ Frequency_tick_check_2, data=surv)
car::S(test_age)
(ci <- confint(test_age))
exp(cbind(OR = coef(test_age), ci))

#### ordinal regression models testing exposure to tick habitat adjusting for gender and
age on survey responses with plots displaying model results
library(gmodels)
library(brant)
library(MASS)
```

```
library(nnet)
library(ggeffects)
library(car)
library(emmeans)
library(forestplot)

### evaluating the association between self-reported exposure to tick habitat and
repellant usage with ORDINAL regression; multinomial regression was tested, and ordinal
had higher AIC
surv$Repellant_use <- relevel(surv$Repellant_use, ref="Never")
gmodels::CrossTable(surv$Exposure_group_binary, surv$Repellant_use) # cross tabulation of
exposure and outcome

## ordinal regression model 1
model1 <- polr(Repellant_use ~ Exposure_group_binary + Age + Gender, data=surv, Hess=TRUE)
# ordinal logistic regression model
car::S(model1) # obtain summary statistics
ci_model1 <- confint(model1) # create confidence intervals
exp_coefs_model1 <- exp(cbind(OR = coef(model1), ci_model1)) # exponentiate
exp_coefs_model1

## marginal effects
pred_test <- ggeffects::ggpredict(model1, c("Exposure_group_binary", "Gender", "Age"))
pred_test_df <- as.data.frame(pred_test)
pred_test_df$response.level <- factor(pred_test_df$response.level, levels = c("Never",
"Sometimes", "Always"))
pred_test_df$x <- recode(pred_test_df$x,
                         "Low exposure to tick habitat" = "Low",
                         "High exposure to tick habitat" = "High")
pred_test_df$x <- factor(pred_test_df$x, levels = c("Never", "Sometimes", "Always"))
View(pred_test_df)

## plot marginal effects
model1_effects <- ggplot(pred_test_df, aes(x = x, y = predicted, color = facet, group =
facet)) +
  geom_point(size = 3) +
  geom_line(size = 1) +
  geom_errorbar(aes(ymin = conf.low, ymax = conf.high), width = 0.2, size = 0.2) +
  facet_grid(group ~ response.level) +
  labs(x = "Self-reported exposure to tick habitat",
       y = "Predicted probability of using repellants",
       color = "Age",
       title = NULL) +
  theme_bw() +
  theme(
    strip.text = element_text(size = 12),
    legend.position = "bottom",
    panel.grid.major.x = element_blank(),
    axis.title.y = element_text(color = "black", size = 14, face = "bold"),
    axis.title.x = element_text(color = "black", size = 14, face = "bold"),
    strip.text.x = element_text(color = "black", size = 14, face = "bold"),
    strip.text.y = element_text(color = "black", size = 14, face = "bold"),
    legend.title = element_text(color = "black", size = 14, face = "bold"),
    legend.text = element_text(color = "black", size = 12),
    axis.text.x = element_text(color = "black", size = 14),
    panel.border = element_rect(color = "black")
  ) +
  scale_color_manual(values = c("gray53", "indianred4", "darkgoldenrod2", "darkblue"))
model1_effects
ggsave(filename = "model1_effects.png", plot = model1_effects,  width = 12, height = 10,
dpi = 600)

### evaluating the association between self-reported exposure to tick habitat and
frequency of ticks on person with ORDINAL regression; multinomial regression was tested,
and ordinal had higher AIC
```

```
surv$Frequency_ticks_on_person <- relevel(surv$Frequency_ticks_on_person, ref="0 times per
week")
gmodels::CrossTable(surv$Exposure_group_binary, surv$Frequency_ticks_on_person) # cross
tabulation of exposure and outcome

## ordinal regression model 2
model2 <- polr(Frequency_ticks_on_person ~ Exposure_group_binary + Age + Gender,
data=surv, Hess=TRUE) # ordinal logistic regression model
car::S(model2) # obtain summary statistics
ci_model2 <- confint(model2) # create confidence intervals
exp_coefs_model2 <- exp(cbind(OR = coef(model2), ci_model2)) # exponentiate
exp_coefs_model2

# test recoded frequency of tick checks
model2_test <- glm(Frequency_ticks_on_person_2 ~ Exposure_group_binary + Age + Gender,
data = surv, family = "binomial")
car::S(model2_test)

## marginal effects
pred_model2 <- ggeffects::ggpredict(model2, c("Exposure_group_binary", "Gender", "Age"))
pred_model2_df <- as.data.frame(pred_model2)
pred_model2_df$response.level <- factor(pred_model2_df$response.level, levels = c("0 times
per week", "1-2 times per week", "> 2 times per week"))
pred_model2_df$x <- recode(pred_model2_df$x,
                           "Low exposure to tick habitat" = "Low",
                           "High exposure to tick habitat" = "High")
head(pred_model2_df)

## plot marginal effects
model2_effects <- ggplot(pred_model2_df, aes(x = x, y = predicted, color = facet, group =
facet)) +
  geom_point(size = 3) +
  geom_line(size = 1) +
  geom_errorbar(aes(ymin = conf.low, ymax = conf.high), width = 0.2, size = 0.2) +
  facet_grid(group ~ response.level) +
  labs(x = "Self-reported exposure to tick habitat",
       y = "Predicted probability of finding ticks on oneself",
       color = "Age",
       title = NULL) +
  theme_bw() +
  theme(
    strip.text = element_text(size = 12),
    legend.position = "bottom",
    panel.grid.major.x = element_blank(),
    axis.title.y = element_text(color = "black", size = 14, face = "bold"),
    axis.title.x = element_text(color = "black", size = 14, face = "bold"),
    strip.text.x = element_text(color = "black", size = 14, face = "bold"),
    strip.text.y = element_text(color = "black", size = 14, face = "bold"),
    legend.title = element_text(color = "black", size = 14, face = "bold"),
    legend.text = element_text(color = "black", size = 12),
    axis.text.x = element_text(color = "black", size = 14),
    panel.border = element_rect(color = "black")
  ) +
  scale_color_manual(values = c("gray53", "indianred4", "darkgoldenrod2", "darkblue"))
model2_effects
ggsave(filename = "model2_effects.png", plot = model2_effects,  width = 12, height = 10,
dpi = 600)


### evaluating the association between self-reported exposure to tick habitat and
frequency of tick checks with ORDINAL regression; multinomial regression was tested, and
ordinal had higher AIC
surv$Frequency_tick_check <- relevel(surv$Frequency_tick_check, ref="Never")
gmodels::CrossTable(surv$Exposure_group_binary, surv$Frequency_tick_check) # cross
tabulation of exposure and outcome
```

```
## ordinal regression model 3
model3 <- polr(Frequency_tick_check ~ Exposure_group_binary + Age + Gender, data=surv,
Hess=TRUE) # ordinal logistic regression model
car::S(model3) # obtain summary statistics - AIC 146.16
ci_model3 <- confint(model3) # create confidence intervals
exp_coefs_model3 <- exp(cbind(OR = coef(model3), ci_model3)) # exponentiate
exp_coefs_model3

# test recoded frequency of tick checks
model3_test <- polr(Frequency_tick_check_2 ~ Exposure_group_binary + Repellant_use_2 +
Age_2 + Gender, data=surv, Hess=TRUE) # ordinal logistic regression model
car::S(model3_test) # obtain summary statistics - AIC 106.55

## marginal effects
pred_model3 <- ggeffects::ggpredict(model3, c("Exposure_group_binary", "Gender", "Age"))
pred_model3_df <- as.data.frame(pred_model3)
pred_model3_df$response.level <- recode(pred_model3_df$response.level,
                                        "Never" = "Never",
                                        "Only when I am in tick habitat" = "In tick
habitat",
                                        "Some days" = "Some days",
                                        "Multiple times a day" = "Multiple per day",
                                        "Every day" = "Every day")
pred_model3_df$response.level <- factor(pred_model3_df$response.level, levels = c("Never",
"In tick habitat", "Some days", "Multiple per day", "Every day"))
pred_model3_df$x <- recode(pred_model3_df$x,
                           "Low exposure to tick habitat" = "Low",
                           "High exposure to tick habitat" = "High")
head(pred_model3_df)

## plot marginal effects
model3_effects <- ggplot(pred_model3_df, aes(x = x, y = predicted, color = facet, group =
facet)) +
  geom_point(size = 3) +
  geom_line(size = 1) +
  geom_errorbar(aes(ymin = conf.low, ymax = conf.high), width = 0.2, size = 0.2) +
  facet_grid(group ~ response.level) +
  labs(x = "Self-reported exposure to tick habitat",
       y = "Predicted probability of performing tick checks",
       color = "Age",
       title = NULL) +
  theme_bw() +
  theme(
    strip.text = element_text(size = 12),
    legend.position = "bottom",
    panel.grid.major.x = element_blank(),
    axis.title.y = element_text(color = "black", size = 14, face = "bold"),
    axis.title.x = element_text(color = "black", size = 14, face = "bold"),
    strip.text.x = element_text(color = "black", size = 14, face = "bold"),
    strip.text.y = element_text(color = "black", size = 14, face = "bold"),
    legend.title = element_text(color = "black", size = 14, face = "bold"),
    legend.text = element_text(color = "black", size = 12),
    axis.text.x = element_text(color = "black", size = 14),
    panel.border = element_rect(color = "black")
  ) +
  scale_color_manual(values = c("gray53", "indianred4", "darkgoldenrod2", "darkblue"))
model3_effects
ggsave(filename = "model3_effects.png", plot = model3_effects,  width = 12, height = 10,
dpi = 600)

### evaluating the association between self-reported exposure to tick habitat and TBD
concern with ORDINAL regression; multinomial regression was tested, and ordinal had higher
AIC
surv$TBD_concern <- relevel(surv$TBD_concern, ref="Not at all concerned")
```

```
gmodels::CrossTable(surv$Exposure_group_binary, surv$TBD_concern) # cross tabulation of
exposure and outcome

## ordinal regression model 4
model4 <- polr(TBD_concern ~ Exposure_group_binary + Age + Gender, data=surv, Hess=TRUE) #
ordinal logistic regression model
car::S(model4) # obtain summary statistics - AIC 135.64
ci_model4 <- confint(model4) # create confidence intervals
exp_coefs_model4 <- exp(cbind(OR = coef(model4), ci_model4)) # exponentiate
exp_coefs_model4

## marginal effects
pred_model4 <- ggeffects::ggpredict(model4, c("Exposure_group_binary", "Gender", "Age"))
pred_model4_df <- as.data.frame(pred_model4)
pred_model4_df$response.level <- recode(pred_model4_df$response.level,
                                        "Not at all concerned" = "Not at all",
                                        "Somewhat concerned" = "Somewhat",
                                        "Moderately concerned" = "Moderately",
                                        "Extremely concerned" = "Extremely")
pred_model4_df$response.level <- factor(pred_model4_df$response.level, levels = c("Not at
all", "Somewhat", "Moderately", "Extremely"))
pred_model4_df$x <- recode(pred_model4_df$x,
                           "Low exposure to tick habitat" = "Low",
                           "High exposure to tick habitat" = "High")
head(pred_model4_df)

## plot marginal effects
model4_effects <- ggplot(pred_model4_df, aes(x = x, y = predicted, color = facet, group =
facet)) +
  geom_point(size = 3) +
  geom_line(size = 1) +
  geom_errorbar(aes(ymin = conf.low, ymax = conf.high), width = 0.2, size = 0.2) +
  facet_grid(group ~ response.level) +
  labs(x = "Self-reported exposure to tick habitat",
       y = "Predicted probability of TBD concern",
       color = "Age",
       title = NULL) +
  theme_bw() +
  theme(
    strip.text = element_text(size = 12),
    legend.position = "bottom",
    panel.grid.major.x = element_blank(),
    axis.title.y = element_text(color = "black", size = 14, face = "bold"),
    axis.title.x = element_text(color = "black", size = 14, face = "bold"),
    strip.text.x = element_text(color = "black", size = 14, face = "bold"),
    strip.text.y = element_text(color = "black", size = 14, face = "bold"),
    legend.title = element_text(color = "black", size = 14, face = "bold"),
    legend.text = element_text(color = "black", size = 12),
    axis.text.x = element_text(color = "black", size = 14),
    panel.border = element_rect(color = "black")
  ) +
  scale_color_manual(values = c("gray53", "indianred4", "darkgoldenrod2", "darkblue"))
model4_effects
ggsave(filename = "model4_effects.png", plot = model4_effects,  width = 12, height = 10,
dpi = 600)

## aggregated marginal effects plot
effects_ordinal <- ggarrange(model1_effects, model2_effects, model3_effects,
model4_effects, ncol=2, nrow=2, common.legend = TRUE, legend = "top")
effects_ordinal_2 <- annotate_figure(effects_ordinal, bottom = text_grob("Self-reported
exposure to tick habitat", size = 14, face = "bold"))
effects_ordinal_2
ggsave(filename = "ordinal_effects.png", plot = effects_ordinal_2,  width = 18, height =
14, dpi = 600)
```

```
#### estimating risk index
## load in tick data
library(readxl)
tick<-read_excel("/Users/jtcassens/Desktop/umn/research/dissertation/aim_1/Data.xlsx",
sheet="Sheet2")
View(tick)
str(tick)
tick$site <- as.factor(tick$site)
tick$habitat_type <- as.factor(tick$habitat_type)
tick$forest_type <- as.factor(tick$forest_type)

median(tick$dit_bb_all_100m[tick$site=="wsh_ct"])
quantile(tick$dit_bb_all_100m[tick$site=="wsh_ct"], 0.25)
quantile(tick$dit_bb_all_100m[tick$site=="wsh_ct"], 0.75)
quantile(tick$dit_bb_all_100m, 0.75)

## define variables for formula
surv$Exposure_fraction_computed[surv$Exposure_fraction_computed ==1 ] <- 0.99999

### WASHINGTON COUNTY
## washington county borrelia burgdorferi NYMPHAL infected tick encounter probability
surv$Pb_Wc_23_Bb_n <- (1-(1-surv$Exposure_fraction_computed)^tick$din_bb_23_100m[tick$site
== "wsh_ct"]) #infected tick encounter probability per 100 m for washington county in 2023
surv$Pb_Wc_24_Bb_n <- (1-(1-surv$Exposure_fraction_computed)^tick$din_bb_24_100m[tick$site
== "wsh_ct"]) #infected tick encounter probability per 100 m for washington county in 2024
surv$Pb_Wc_total_Bb_n <- (1-(1-
surv$Exposure_fraction_computed)^tick$din_bb_all_100m[tick$site == "wsh_ct"]) #infected
tick encounter probability per 100 m for washington county in total

## washington county anaplasma phagocytophilum NYMPHAL infected tick encounter probability
surv$Pb_Wc_23_Ap_n <- (1-(1-surv$Exposure_fraction_computed)^tick$din_ap_23_100m[tick$site
== "wsh_ct"]) #infected tick encounter probability per 100 m for washington county in 2023
surv$Pb_Wc_24_Ap_n <- (1-(1-surv$Exposure_fraction_computed)^tick$din_ap_24_100m[tick$site
== "wsh_ct"]) #infected tick encounter probability per 100 m for washington county in 2024
surv$Pb_Wc_total_Ap_n <- (1-(1-
surv$Exposure_fraction_computed)^tick$din_ap_all_100m[tick$site == "wsh_ct"]) #infected
tick encounter probability per 100 m for washington county in total

## washington county borrelia burgdorferi ADULT infected tick encounter probability
surv$Pb_Wc_23_Bb_a <- (1-(1-surv$Exposure_fraction_computed)^tick$dia_bb_23_100m[tick$site
== "wsh_ct"]) #infected tick encounter probability per 100 m for washington county in 2023
surv$Pb_Wc_24_Bb_a <- (1-(1-surv$Exposure_fraction_computed)^tick$dia_bb_24_100m[tick$site
== "wsh_ct"]) #infected tick encounter probability per 100 m for washington county in 2024
surv$Pb_Wc_total_Bb_a <- (1-(1-
surv$Exposure_fraction_computed)^tick$dia_bb_all_100m[tick$site == "wsh_ct"]) #infected
tick encounter probability per 100 m for washington county in total

## washington county anaplasma phagocytophilum ADULT infected tick encounter probability
surv$Pb_Wc_23_Ap_a <- (1-(1-surv$Exposure_fraction_computed)^tick$dia_ap_23_100m[tick$site
== "wsh_ct"]) #infected tick encounter probability per 100 m for washington county in 2023
surv$Pb_Wc_24_Ap_a <- (1-(1-surv$Exposure_fraction_computed)^tick$dia_ap_24_100m[tick$site
== "wsh_ct"]) #infected tick encounter probability per 100 m for washington county in 2024
surv$Pb_Wc_total_Ap_a <- (1-(1-
surv$Exposure_fraction_computed)^tick$dia_ap_all_100m[tick$site == "wsh_ct"]) #infected
tick encounter probability per 100 m for washington county in total

## washington county borrelia burgdorferi TOTAL infected tick encounter probability
surv$Pb_Wc_23_Bb_t <- (1-(1-surv$Exposure_fraction_computed)^tick$dit_bb_23_100m[tick$site
== "wsh_ct"]) #infected tick encounter probability per 100 m for washington county in 2023
surv$Pb_Wc_24_Bb_t <- (1-(1-surv$Exposure_fraction_computed)^tick$dit_bb_24_100m[tick$site
== "wsh_ct"]) #infected tick encounter probability per 100 m for washington county in 2024
surv$Pb_Wc_total_Bb_t <- (1-(1-
surv$Exposure_fraction_computed)^tick$dit_bb_all_100m[tick$site == "wsh_ct"]) #infected
tick encounter probability per 100 m for washington county in total
```

```
## washington county anaplasma phagocytophilum TOTAL infected tick encounter probability
surv$Pb_Wc_23_Ap_t <- (1-(1-surv$Exposure_fraction_computed)^tick$dit_ap_23_100m[tick$site
== "wsh_ct"]) #infected tick encounter probability per 100 m for washington county in 2023
surv$Pb_Wc_24_Ap_t <- (1-(1-surv$Exposure_fraction_computed)^tick$dit_ap_24_100m[tick$site
== "wsh_ct"]) #infected tick encounter probability per 100 m for washington county in 2024
surv$Pb_Wc_total_Ap_t <- (1-(1-
surv$Exposure_fraction_computed)^tick$dit_ap_all_100m[tick$site == "wsh_ct"]) #infected
tick encounter probability per 100 m for washington county in total

### CARLOS AVERY
## carlos avery borrelia burgdorferi infected NYMPHAL tick encounter probability
surv$Pb_Ca_23_Bb_n <- (1-(1-surv$Exposure_fraction_computed)^tick$din_bb_23_100m[tick$site
== "carlos_av"]) #infected tick encounter probability per 100 m for carlos avery in 2023
surv$Pb_Ca_24_Bb_n <- (1-(1-surv$Exposure_fraction_computed)^tick$din_bb_24_100m[tick$site
== "carlos_av"]) #infected tick encounter probability per 100 m for carlos avery in 2024
surv$Pb_Ca_total_Bb_n <- (1-(1-
surv$Exposure_fraction_computed)^tick$din_bb_all_100m[tick$site == "carlos_av"]) #infected
tick encounter probability per 100 m for carlos avery in total

## carlos avery anaplasma phagocytophilum NYMPHAL infected tick encounter probability
surv$Pb_Ca_23_Ap_n <- (1-(1-surv$Exposure_fraction_computed)^tick$din_ap_23_100m[tick$site
== "carlos_av"]) #infected tick encounter probability per 100 m for carlos avery in 2023
surv$Pb_Ca_24_Ap_n <- (1-(1-surv$Exposure_fraction_computed)^tick$din_ap_24_100m[tick$site
== "carlos_av"]) #infected tick encounter probability per 100 m for carlos avery in 2024
surv$Pb_Ca_total_Ap_n <- (1-(1-
surv$Exposure_fraction_computed)^tick$din_ap_all_100m[tick$site == "carlos_av"]) #infected
tick encounter probability per 100 m for carlos avery in total

## carlos avery borrelia burgdorferi infected ADULT tick encounter probability
surv$Pb_Ca_23_Bb_a <- (1-(1-surv$Exposure_fraction_computed)^tick$dia_bb_23_100m[tick$site
== "carlos_av"]) #infected tick encounter probability per 100 m for carlos avery in 2023
surv$Pb_Ca_24_Bb_a <- (1-(1-surv$Exposure_fraction_computed)^tick$dia_bb_24_100m[tick$site
== "carlos_av"]) #infected tick encounter probability per 100 m for carlos avery in 2024
surv$Pb_Ca_total_Bb_a <- (1-(1-
surv$Exposure_fraction_computed)^tick$dia_bb_all_100m[tick$site == "carlos_av"]) #infected
tick encounter probability per 100 m for carlos avery in total

## carlos avery anaplasma phagocytophilum ADULT infected tick encounter probability
surv$Pb_Ca_23_Ap_a <- (1-(1-surv$Exposure_fraction_computed)^tick$dia_ap_23_100m[tick$site
== "carlos_av"]) #infected tick encounter probability per 100 m for carlos avery in 2023
surv$Pb_Ca_24_Ap_a <- (1-(1-surv$Exposure_fraction_computed)^tick$dia_ap_24_100m[tick$site
== "carlos_av"]) #infected tick encounter probability per 100 m for carlos avery in 2024
surv$Pb_Ca_total_Ap_a <- (1-(1-
surv$Exposure_fraction_computed)^tick$dia_ap_all_100m[tick$site == "carlos_av"]) #infected
tick encounter probability per 100 m for carlos avery in total

## carlos avery borrelia burgdorferi infected TOTAL tick encounter probability
surv$Pb_Ca_23_Bb_t <- (1-(1-surv$Exposure_fraction_computed)^tick$dit_bb_23_100m[tick$site
== "carlos_av"]) #infected tick encounter probability per 100 m for carlos avery in 2023
surv$Pb_Ca_24_Bb_t <- (1-(1-surv$Exposure_fraction_computed)^tick$dit_bb_24_100m[tick$site
== "carlos_av"]) #infected tick encounter probability per 100 m for carlos avery in 2024
surv$Pb_Ca_total_Bb_t <- (1-(1-
surv$Exposure_fraction_computed)^tick$dit_bb_all_100m[tick$site == "carlos_av"]) #infected
tick encounter probability per 100 m for carlos avery in total

## carlos avery anaplasma phagocytophilum TOTAL infected tick encounter probability
surv$Pb_Ca_23_Ap_t <- (1-(1-surv$Exposure_fraction_computed)^tick$dit_ap_23_100m[tick$site
== "carlos_av"]) #infected tick encounter probability per 100 m for carlos avery in 2023
surv$Pb_Ca_24_Ap_t <- (1-(1-surv$Exposure_fraction_computed)^tick$dit_ap_24_100m[tick$site
== "carlos_av"]) #infected tick encounter probability per 100 m for carlos avery in 2024
surv$Pb_Ca_total_Ap_t <- (1-(1-
surv$Exposure_fraction_computed)^tick$dit_ap_all_100m[tick$site == "carlos_av"]) #infected
tick encounter probability per 100 m for carlos avery in total
```

```
### WHITEWATER
## whitewater borrelia burgdorferi infected NYMPHAL tick encounter probability
surv$Pb_Ww_23_Bb_n <- (1-(1-surv$Exposure_fraction_computed)^tick$din_bb_23_100m[tick$site
== "whitewater"]) #infected tick encounter probability per 100 m for whitewater in 2023
surv$Pb_Ww_24_Bb_n <- (1-(1-surv$Exposure_fraction_computed)^tick$din_bb_24_100m[tick$site
== "whitewater"]) #infected tick encounter probability per 100 m for whitewater in 2024
surv$Pb_Ww_total_Bb_n <- (1-(1-
surv$Exposure_fraction_computed)^tick$din_bb_all_100m[tick$site == "whitewater"])
#infected tick encounter probability per 100 m for whitewater in total

## whitewater anaplasma phagocytophilum infected NYMPHAL tick encounter probability
surv$Pb_Ww_23_Ap_n <- (1-(1-surv$Exposure_fraction_computed)^tick$din_ap_23_100m[tick$site
== "whitewater"]) #infected tick encounter probability per 100 m for whitewater in 2023
surv$Pb_Ww_24_Ap_n <- (1-(1-surv$Exposure_fraction_computed)^tick$din_ap_24_100m[tick$site
== "whitewater"]) #infected tick encounter probability per 100 m for whitewater in 2024
surv$Pb_Ww_total_Ap_n <- (1-(1-
surv$Exposure_fraction_computed)^tick$din_ap_all_100m[tick$site == "whitewater"])
#infected tick encounter probability per 100 m for whitewater in total

## whitewater borrelia burgdorferi infected ADULT tick encounter probability
surv$Pb_Ww_23_Bb_a <- (1-(1-surv$Exposure_fraction_computed)^tick$dia_bb_23_100m[tick$site
== "whitewater"]) #infected tick encounter probability per 100 m for whitewater in 2023
surv$Pb_Ww_24_Bb_a <- (1-(1-surv$Exposure_fraction_computed)^tick$dia_bb_24_100m[tick$site
== "whitewater"]) #infected tick encounter probability per 100 m for whitewater in 2024
surv$Pb_Ww_total_Bb_a <- (1-(1-
surv$Exposure_fraction_computed)^tick$dia_bb_all_100m[tick$site == "whitewater"])
#infected tick encounter probability per 100 m for whitewater in total

## whitewater anaplasma phagocytophilum infected ADULT tick encounter probability
surv$Pb_Ww_23_Ap_a <- (1-(1-surv$Exposure_fraction_computed)^tick$dia_ap_23_100m[tick$site
== "whitewater"]) #infected tick encounter probability per 100 m for whitewater in 2023
surv$Pb_Ww_24_Ap_a <- (1-(1-surv$Exposure_fraction_computed)^tick$dia_ap_24_100m[tick$site
== "whitewater"]) #infected tick encounter probability per 100 m for whitewater in 2024
surv$Pb_Ww_total_Ap_a <- (1-(1-
surv$Exposure_fraction_computed)^tick$dia_ap_all_100m[tick$site == "whitewater"])
#infected tick encounter probability per 100 m for whitewater in total

## whitewater borrelia burgdorferi infected TOTAL tick encounter probability
surv$Pb_Ww_23_Bb_t <- (1-(1-surv$Exposure_fraction_computed)^tick$dit_bb_23_100m[tick$site
== "whitewater"]) #infected tick encounter probability per 100 m for whitewater in 2023
surv$Pb_Ww_24_Bb_t <- (1-(1-surv$Exposure_fraction_computed)^tick$dit_bb_24_100m[tick$site
== "whitewater"]) #infected tick encounter probability per 100 m for whitewater in 2024
surv$Pb_Ww_total_Bb_t <- (1-(1-
surv$Exposure_fraction_computed)^tick$dit_bb_all_100m[tick$site == "whitewater"])
#infected tick encounter probability per 100 m for whitewater in total

## whitewater anaplasma phagocytophilum infected TOTAL tick encounter probability
surv$Pb_Ww_23_Ap_t <- (1-(1-surv$Exposure_fraction_computed)^tick$dit_ap_23_100m[tick$site
== "whitewater"]) #infected tick encounter probability per 100 m for whitewater in 2023
surv$Pb_Ww_24_Ap_t <- (1-(1-surv$Exposure_fraction_computed)^tick$dit_ap_24_100m[tick$site
== "whitewater"]) #infected tick encounter probability per 100 m for whitewater in 2024
surv$Pb_Ww_total_Ap_t <- (1-(1-
surv$Exposure_fraction_computed)^tick$dit_ap_all_100m[tick$site == "whitewater"])
#infected tick encounter probability per 100 m for whitewater in total

### computing infected TOTAL tick encounter probability averaged across all sites
## Borrelia
surv$Pb_all_total_Bb_t <- rowMeans(surv[, c("Pb_Wc_total_Bb_t", "Pb_Ca_total_Bb_t",
"Pb_Ww_total_Bb_t")], na.rm = TRUE)

## anaplasma
surv$Pb_all_total_Ap_t <- rowMeans(surv[, c("Pb_Wc_total_Ap_t", "Pb_Ca_total_Ap_t",
"Pb_Ww_total_Ap_t")], na.rm = TRUE)

View(surv)
```

```
### create boxplot figure to display predicted probability of encountering infected ticks
per week by site and tick lifestage
## reshape dataset to long format for figure production
# reshape bb dataset
library(dplyr)
library(tidyr)
long_surv_bb <- surv %>%
  select(Participant, Pb_Ww_total_Bb_n, Pb_Ww_total_Bb_a, Pb_Ww_total_Bb_t,
         Pb_Ca_total_Bb_n, Pb_Ca_total_Bb_a, Pb_Ca_total_Bb_t,
         Pb_Wc_total_Bb_n, Pb_Wc_total_Bb_a, Pb_Wc_total_Bb_t) %>%
  pivot_longer(
    cols = starts_with("Pb_"),
    names_to = c("Site", "Type"),
    names_pattern = "Pb_(Ww|Ca|Wc)_total_Bb_(n|a|t)",
    values_to = "Predicted_Prob"
  ) %>%
  mutate(Pathogen = "Bb")

# reshape ap dataset
long_surv_ap <- surv %>%
  select(Participant, Pb_Ww_total_Ap_n, Pb_Ww_total_Ap_a, Pb_Ww_total_Ap_t,
         Pb_Ca_total_Ap_n, Pb_Ca_total_Ap_a, Pb_Ca_total_Ap_t,
         Pb_Wc_total_Ap_n, Pb_Wc_total_Ap_a, Pb_Wc_total_Ap_t) %>%
  pivot_longer(
    cols = starts_with("Pb_"),
    names_to = c("Site", "Type"),
    names_pattern = "Pb_(Ww|Ca|Wc)_total_Ap_(n|a|t)",
    values_to = "Predicted_Prob"
  ) %>%
  mutate(Pathogen = "Ap")

# combine both long datasets
combined_data <- bind_rows(long_surv_bb, long_surv_ap)
combined_data$Pathogen <- factor(combined_data$Pathogen, levels = c("Bb", "Ap"))
combined_data$Type <- factor(combined_data$Type, levels = c("n", "a", "t"))

# plot the data
library(ggplot2)
mcohs_figure4 <- ggplot(combined_data, aes(x = Type, y = Predicted_Prob, fill = Type)) +
  geom_boxplot() +
  facet_grid(Pathogen ~ Site, scales = "free_y",
             labeller = labeller(Pathogen = c(Bb = "Borrelia burgdorferi",
                                              Ap = "Anaplasma phagocytophilum"),
                                 Site = c(Ca = "Carlos Avery WMA",
                                          Wc = "Lake Elmo Park Reserve",
                                          Ww = "Whitewater WMA"))) +
  labs(title = NULL,
       x = NULL,
       y = "Predicted probability (per week)",
       fill = "Life stage") +
  scale_y_continuous(labels = scales::percent_format(accuracy = 1),
                     expand = expansion(mult = c(0, 0.05)),
                     limits = c(0,1)) +
  scale_fill_manual(values = c("gray53", "indianred4", "darkgoldenrod2"),
                    labels = c("Nymph", "Adult", "Total")) +
  theme_bw() +
  theme(axis.text.x = element_blank(),
        axis.ticks.x = element_blank(),
        strip.text = element_text(size = 12),
        axis.title.y = element_text(color = "black", size = 14, face = "bold"),
        axis.text = element_text(color = "black", size = 14),
        strip.text.x = element_text(color = "black", size = 14, face = "bold"),
        strip.text.y = element_text(color = "black", size = 14, face = "bold.italic"),
        legend.title = element_text(color = "black", size = 14, face = "bold"),
        legend.text = element_text(color = "black", size = 12),
```

```
        panel.grid.major.x = element_blank(),
        panel.grid.minor.x = element_blank(),
        panel.grid.major.y = element_line(color = "gray90"),
        panel.grid.minor.y = element_line(color = "gray90"),
        panel.border = element_rect(color = "black", fill = NA, linewidth = 0.5),
        panel.spacing = unit(0.3, "lines"),
        panel.spacing.y = unit(1, "lines"),
        plot.background = element_rect(fill = 'white', color = NA))
mcohs_figure4
ggsave(filename = "mcohs_Figure4.png", plot = mcohs_figure4,  width = 9.5, height = 7, dpi
= 600)


#### generalized linear models to predict the probability of infected tick encounters
library(MASS)
library(emmeans)

### Washington county - Lake Elmo park reserve - total
## Borrelia - nymphal ticks
model5 <- glm(Pb_Wc_total_Bb_n ~ Gender + Age + Employment + Repellant_use +
Frequency_ticks_on_person + Frequency_tick_check + TBD_concern, data=surv)
car::S(model5) # summarize full model
(ci <- confint(model5)) # create confidence intervals
exp(cbind(OR = coef(model5), ci)) # exponentiate
step_model5 <- step(model5, direction="both") # stepwise model selection to determine the
best model to predict the risk of infected tick encounters
model5_best <- glm(Pb_Wc_total_Bb_n ~ Age + Frequency_tick_check, data=surv) # best model
as predicted by stepwise selection
car::S(model5_best) # summary of best model
(ci <- confint(model5_best)) # create confidence intervals
exp(cbind(OR = coef(model5_best), ci)) # exponentiate
ggeffects::ggpredict(model5_best, c("Frequency_tick_check", "Age"))

mean(surv$Pb_Wc_total_Bb_n)

## Anaplasma - nymphal ticks
model6 <- glm(Pb_Wc_total_Ap_n ~ Gender + Age + Employment + Repellant_use +
Frequency_ticks_on_person + Frequency_tick_check + TBD_concern, data=surv)
car::S(model6) # summarize full model
(ci <- confint(model6)) # create confidence intervals
exp(cbind(OR = coef(model6), ci)) # exponentiate
step_model6 <- step(model6, direction="both") # stepwise model selection to determine the
best model to predict the risk of infected tick encounters
model6_best <- glm(Pb_Wc_total_Ap_n ~ Age + Frequency_tick_check, data=surv) # best model
as predicted by stepwise selection
car::S(model6_best) # summary of best model
(ci <- confint(model6_best)) # create confidence intervals
exp(cbind(OR = coef(model6_best), ci)) # exponentiate
ggeffects::ggpredict(model6_best, c("Frequency_tick_check", "Age"))

## Borrelia - adult ticks
model7 <- glm(Pb_Wc_total_Bb_a ~ Gender + Age + Employment + Repellant_use +
Frequency_ticks_on_person + Frequency_tick_check + TBD_concern, data=surv)
car::S(model7) # summarize full model
(ci <- confint(model7)) # create confidence intervals
exp(cbind(OR = coef(model7), ci)) # exponentiate
step_model7 <- step(model7, direction="both") # stepwise model selection to determine the
best model to predict the risk of infected tick encounters
model7_best <- glm(Pb_Wc_total_Bb_a ~ Age + Frequency_tick_check, data=surv) # best model
as predicted by stepwise selection
car::S(model7_best) # summary of best model
(ci <- confint(model7_best)) # create confidence intervals
exp(cbind(OR = coef(model7_best), ci)) # exponentiate
ggeffects::ggpredict(model7_best, c("Frequency_tick_check", "Age"))
```

```
mean(surv$Pb_Wc_total_Bb_a)

## Anaplasma - adult ticks
model8 <- glm(Pb_Wc_total_Ap_a ~ Gender + Age + Employment + Repellant_use +
Frequency_ticks_on_person + Frequency_tick_check + TBD_concern, data=surv)
car::S(model8) # summarize full model
(ci <- confint(model8)) # create confidence intervals
exp(cbind(OR = coef(model8), ci)) # exponentiate
step_model8 <- step(model8, direction="both") # stepwise model selection to determine the
best model to predict the risk of infected tick encounters
model8_best <- glm(Pb_Wc_total_Ap_a ~ Age + Frequency_tick_check, data=surv) # best model
as predicted by stepwise selection
car::S(model8_best) # summary of best model
(ci <- confint(model8_best)) # create confidence intervals
exp(cbind(OR = coef(model8_best), ci)) # exponentiate
ggeffects::ggpredict(model8_best, c("Frequency_tick_check", "Age"))

## Borrelia - total ticks
model9 <- glm(Pb_Wc_total_Bb_t ~ Gender + Age + Employment + Repellant_use +
Frequency_ticks_on_person + Frequency_tick_check_2 + TBD_concern, data=surv)
car::S(model9) # summarize full model - AIC 5.37
(ci <- confint(model9)) # create confidence intervals
exp(cbind(OR = coef(model9), ci)) # exponentiate
step_model9 <- step(model9, direction="both") # stepwise model selection to determine the
best model to predict the risk of infected tick encounters
model9_best <- glm(Pb_Wc_total_Bb_t ~ Age, data=surv) # best model as predicted by
stepwise selection
car::S(model9_best) # summary of best model - AIC -9.31
(ci <- confint(model9_best)) # create confidence intervals
exp(cbind(OR = coef(model9_best), ci)) # exponentiate
ggeffects::ggpredict(model9_best, c("Frequency_tick_check", "Age"))
Wc_Bb_pp_effects <- plot(ggeffects::ggpredict(model9_best, c("Frequency_tick_check",
"Age")))

summary(surv$Pb_Wc_total_Bb_t)

# test with multi-model inference selection
library(MuMIn)
model9_test <- glm(Pb_Wc_total_Bb_t ~ Gender + Age + Employment + Repellant_use +
Frequency_ticks_on_person + Frequency_tick_check + TBD_concern, data=surv, na.action =
na.fail)
model_set9_test <- dredge(model9_test)
avg_model_set9_test <- model.avg(model_set9_test, subset = delta < 4)
car::S(avg_model_set9_test)
(ci <- confint(avg_model_set9_test, full = TRUE)) # create confidence intervals
exp(cbind(OR = coef(avg_model_set9_test, full = TRUE), ci)) # exponentiate
selected_model9 <- get.models(model_set9_test, subset = delta < 4)


## Anaplasma - total ticks
model10 <- glm(Pb_Wc_total_Ap_t ~ Gender + Age + Employment + Repellant_use +
Frequency_ticks_on_person + Frequency_tick_check + TBD_concern, data=surv)
car::S(model10) # summarize full model
(ci <- confint(model10)) # create confidence intervals
exp(cbind(OR = coef(model10), ci)) # exponentiate
step_model10 <- step(model10, direction="both") # stepwise model selection to determine
the best model to predict the risk of infected tick encounters
model10_best <- glm(Pb_Wc_total_Ap_t ~ Age + Frequency_tick_check, data=surv) # best model
as predicted by stepwise selection
car::S(model10_best) # summary of best model
(ci <- confint(model10_best)) # create confidence intervals
exp(cbind(OR = coef(model10_best), ci)) # exponentiate
ggeffects::ggpredict(model10_best, c("Frequency_tick_check", "Age"))
Wc_Ap_pp_effects <- plot(ggeffects::ggpredict(model10_best, c("Frequency_tick_check",
"Age")))
```

```
# test with multi-model inference selection
model10_test <- glm(Pb_Wc_total_Ap_t ~ Gender + Age + Employment + Repellant_use +
Frequency_ticks_on_person + Frequency_tick_check + TBD_concern, data=surv, na.action =
na.fail)
model_set10_test <- dredge(model10_test)
avg_model_set10_test <- model.avg(model_set10_test, subset = delta < 4)
car::S(avg_model_set10_test)
(ci <- confint(avg_model_set10_test, full=TRUE)) # create confidence intervals
exp(cbind(OR = coef(avg_model_set10_test, full=TRUE), ci)) # exponentiate
selected_model10 <- get.models(model_set10_test, subset = delta < 4)


### Carlos Avery wildlife management area - total
## Borrelia - nymphal ticks
model11 <- glm(Pb_Ca_total_Bb_n ~ Gender + Age + Employment + Repellant_use +
Frequency_ticks_on_person + Frequency_tick_check + TBD_concern, data=surv)
car::S(model11) # summarize full model
(ci <- confint(model11)) # create confidence intervals
exp(cbind(OR = coef(model11), ci)) # exponentiate
step_model11 <- step(model11, direction="both") # stepwise model selection to determine
the best model to predict the risk of infected tick encounters
model11_best <- glm(Pb_Ca_total_Bb_n ~ Age + Frequency_tick_check, data=surv) # best model
as predicted by stepwise selection
car::S(model11_best) # summary of best model
(ci <- confint(model11_best)) # create confidence intervals
exp(cbind(OR = coef(model11_best), ci)) # exponentiate
ggeffects::ggpredict(model11_best, c("Frequency_tick_check", "Age"))

summary(surv$Pb_Ca_total_Bb_n)

## Anaplasma - nymphal ticks
model12 <- glm(Pb_Ca_total_Ap_n ~ Gender + Age + Employment + Repellant_use +
Frequency_ticks_on_person + Frequency_tick_check + TBD_concern, data=surv)
car::S(model12) # summarize full model
(ci <- confint(model12)) # create confidence intervals
exp(cbind(OR = coef(model12), ci)) # exponentiate
step_model12 <- step(model12, direction="both") # stepwise model selection to determine
the best model to predict the risk of infected tick encounters
model12_best <- glm(Pb_Ca_total_Ap_n ~ Age + Frequency_tick_check, data=surv) # best model
as predicted by stepwise selection
car::S(model12_best) # summary of best model
(ci <- confint(model12_best)) # create confidence intervals
exp(cbind(OR = coef(model12_best), ci)) # exponentiate
ggeffects::ggpredict(model12_best, c("Frequency_tick_check", "Age"))

## Borrelia - adult ticks
model13 <- glm(Pb_Ca_total_Bb_a ~ Gender + Age + Employment + Repellant_use +
Frequency_ticks_on_person + Frequency_tick_check + TBD_concern, data=surv)
car::S(model13) # summarize full model
(ci <- confint(model13)) # create confidence intervals
exp(cbind(OR = coef(model13), ci)) # exponentiate
step_model13 <- step(model13, direction="both") # stepwise model selection to determine
the best model to predict the risk of infected tick encounters
model13_best <- glm(Pb_Ca_total_Bb_a ~ Age + Frequency_tick_check, data=surv) # best model
as predicted by stepwise selection
car::S(model13_best) # summary of best model
(ci <- confint(model13_best)) # create confidence intervals
exp(cbind(OR = coef(model13_best), ci)) # exponentiate
ggeffects::ggpredict(model13_best, c("Frequency_tick_check", "Age"))

summary(surv$Pb_Ca_total_Bb_a)

## Anaplasma - adult ticks
model14 <- glm(Pb_Ca_total_Ap_a ~ Gender + Age + Employment + Repellant_use +
```

```
Frequency_ticks_on_person + Frequency_tick_check + TBD_concern, data=surv)
car::S(model14) # summarize full model
(ci <- confint(model14)) # create confidence intervals
exp(cbind(OR = coef(model14), ci)) # exponentiate
step_model14 <- step(model14, direction="both") # stepwise model selection to determine
the best model to predict the risk of infected tick encounters
model14_best <- glm(Pb_Ca_total_Ap_a ~ Age + Frequency_tick_check, data=surv) # best model
as predicted by stepwise selection
car::S(model14_best) # summary of best model
(ci <- confint(model14_best)) # create confidence intervals
exp(cbind(OR = coef(model14_best), ci)) # exponentiate
ggeffects::ggpredict(model14_best, c("Frequency_tick_check", "Age"))

## Borrelia - total ticks
model15 <- glm(Pb_Ca_total_Bb_t ~ Gender + Age + Employment + Repellant_use +
Frequency_ticks_on_person + Frequency_tick_check + TBD_concern, data=surv)
car::S(model15) # summarize full model
(ci <- confint(model15)) # create confidence intervals
exp(cbind(OR = coef(model15), ci)) # exponentiate
step_model15 <- step(model15, direction="both") # stepwise model selection to determine
the best model to predict the risk of infected tick encounters
model15_best <- glm(Pb_Ca_total_Bb_t ~ Age + Frequency_tick_check_2, data=surv) # best
model as predicted by stepwise selection
car::S(model15_best) # summary of best model
(ci <- confint(model15_best)) # create confidence intervals
exp(cbind(OR = coef(model15_best), ci)) # exponentiate
ggeffects::ggpredict(model15_best, c("Frequency_tick_check", "Age"))
Ca_Bb_pp_effects <- plot(ggeffects::ggpredict(model15_best, c("Frequency_tick_check",
"Age")))

summary(surv$Pb_Ca_total_Bb_t)

# test with multi-model inference selection
model15_test <- glm(Pb_Ca_total_Bb_t ~ Gender + Age + Employment + Repellant_use +
Frequency_ticks_on_person + Frequency_tick_check + TBD_concern, data=surv, na.action =
na.fail)
model_set15_test <- dredge(model15_test)
avg_model_set15_test <- model.avg(model_set15_test, subset = delta < 4)
car::S(avg_model_set15_test)
(ci <- confint(avg_model_set15_test, full=TRUE)) # create confidence intervals
exp(cbind(OR = coef(avg_model_set15_test, full=TRUE), ci)) # exponentiate
selected_model9 <- get.models(model_set9_test, subset = delta < 4)

## Anaplasma - total ticks
model16 <- glm(Pb_Ca_total_Ap_t ~ Gender + Age + Employment + Repellant_use +
Frequency_ticks_on_person + Frequency_tick_check + TBD_concern, data=surv)
car::S(model16) # summarize full model
(ci <- confint(model16)) # create confidence intervals
exp(cbind(OR = coef(model16), ci)) # exponentiate
step_model16 <- step(model16, direction="both") # stepwise model selection to determine
the best model to predict the risk of infected tick encounters
model16_best <- glm(Pb_Ca_total_Ap_t ~ Age + Frequency_tick_check, data=surv) # best model
as predicted by stepwise selection
car::S(model16_best) # summary of best model
(ci <- confint(model16_best)) # create confidence intervals
exp(cbind(OR = coef(model16_best), ci)) # exponentiate
ggeffects::ggpredict(model16_best, c("Frequency_tick_check", "Age"))
Ca_Ap_pp_effects <- plot(ggeffects::ggpredict(model16_best, c("Frequency_tick_check",
"Age")))

summary(surv$Pb_Ca_total_Ap_t)

# test with multi-model inference selection
model16_test <- glm(Pb_Ca_total_Ap_t ~ Gender + Age + Employment + Repellant_use +
Frequency_ticks_on_person + Frequency_tick_check + TBD_concern, data=surv, na.action =
```

```
na.fail)
model_set16_test <- dredge(model16_test)
avg_model_set16_test <- model.avg(model_set16_test, subset = delta < 4)
car::S(avg_model_set16_test)
(ci <- confint(avg_model_set16_test, full=TRUE)) # create confidence intervals
exp(cbind(OR = coef(avg_model_set16_test, full=TRUE), ci)) # exponentiate
selected_model16 <- get.models(model_set16_test, subset = delta < 4)


### Whitewater wildlife management area - total
## Borrelia - nymphal ticks
model17 <- glm(Pb_Ww_total_Bb_n ~ Gender + Age + Employment + Repellant_use +
Frequency_ticks_on_person + Frequency_tick_check + TBD_concern, data=surv)
car::S(model17) # summarize full model
(ci <- confint(model17)) # create confidence intervals
exp(cbind(OR = coef(model17), ci)) # exponentiate
step_model17 <- step(model17, direction="both") # stepwise model selection to determine
the best model to predict the risk of infected tick encounters
model17_best <- glm(Pb_Ww_total_Bb_n ~ Age + Frequency_tick_check, data=surv) # best model
as predicted by stepwise selection
car::S(model17_best) # summary of best model
(ci <- confint(model17_best)) # create confidence intervals
exp(cbind(OR = coef(model17_best), ci)) # exponentiate
ggeffects::ggpredict(model17_best, c("Frequency_tick_check", "Age"))

## Anaplasma - nymphal ticks
model18 <- glm(Pb_Ww_total_Ap_n ~ Gender + Age + Employment + Repellant_use +
Frequency_ticks_on_person + Frequency_tick_check + TBD_concern, data=surv)
car::S(model18) # summarize full model
(ci <- confint(model18)) # create confidence intervals
exp(cbind(OR = coef(model18), ci)) # exponentiate
step_model18 <- step(model18, direction="both") # stepwise model selection to determine
the best model to predict the risk of infected tick encounters
model18_best <- glm(Pb_Ww_total_Ap_n ~ Age + Frequency_tick_check, data=surv) # best model
as predicted by stepwise selection
car::S(model18_best) # summary of best model
(ci <- confint(model18_best)) # create confidence intervals
exp(cbind(OR = coef(model18_best), ci)) # exponentiate
ggeffects::ggpredict(model18_best, c("Frequency_tick_check", "Age"))

## Borrelia - adult ticks
model19 <- glm(Pb_Ww_total_Bb_a ~ Gender + Age + Employment + Repellant_use +
Frequency_ticks_on_person + Frequency_tick_check + TBD_concern, data=surv)
car::S(model19) # summarize full model
(ci <- confint(model19)) # create confidence intervals
exp(cbind(OR = coef(model19), ci)) # exponentiate
step_model19 <- step(model19, direction="both") # stepwise model selection to determine
the best model to predict the risk of infected tick encounters
model19_best <- glm(Pb_Ww_total_Bb_a ~ Age + Frequency_tick_check, data=surv) # best model
as predicted by stepwise selection
car::S(model19_best) # summary of best model
(ci <- confint(model19_best)) # create confidence intervals
exp(cbind(OR = coef(model19_best), ci)) # exponentiate
ggeffects::ggpredict(model19_best, c("Frequency_tick_check", "Age"))

## Anaplasma - adult ticks
model20 <- glm(Pb_Ww_total_Ap_a ~ Gender + Age + Employment + Repellant_use +
Frequency_ticks_on_person + Frequency_tick_check + TBD_concern, data=surv)
car::S(model20) # summarize full model
(ci <- confint(model20)) # create confidence intervals
exp(cbind(OR = coef(model20), ci)) # exponentiate
step_model20 <- step(model20, direction="both") # stepwise model selection to determine
the best model to predict the risk of infected tick encounters
model20_best <- glm(Pb_Ww_total_Ap_a ~ Age + Frequency_tick_check, data=surv) # best model
as predicted by stepwise selection
```

```
car::S(model20_best) # summary of best model
(ci <- confint(model20_best)) # create confidence intervals
exp(cbind(OR = coef(model20_best), ci)) # exponentiate
ggeffects::ggpredict(model20_best, c("Frequency_tick_check", "Age"))

## Borrelia - total ticks
model21 <- glm(Pb_Ww_total_Bb_t ~ Gender + Age + Employment + Repellant_use +
Frequency_ticks_on_person + Frequency_tick_check + TBD_concern, data=surv)
car::S(model21) # summarize full model
(ci <- confint(model21)) # create confidence intervals
exp(cbind(OR = coef(model21), ci)) # exponentiate
step_model21 <- step(model21, direction="both") # stepwise model selection to determine
the best model to predict the risk of infected tick encounters
model21_best <- glm(Pb_Ww_total_Bb_t ~ Age + Frequency_tick_check, data=surv) # best model
as predicted by stepwise selection
car::S(model21_best) # summary of best model
(ci <- confint(model21_best)) # create confidence intervals
exp(cbind(OR = coef(model21_best), ci)) # exponentiate
ggeffects::ggpredict(model21, c("Frequency_tick_check", "Age"))
Ww_Bb_pp_effects <- plot(ggeffects::ggpredict(model21_best, c("Frequency_tick_check",
"Age")))

summary(surv$Pb_Ww_total_Bb_t)

# test with multi-model inference selection
model21_test <- glm(Pb_Ww_total_Bb_t ~ Gender + Age + Employment + Repellant_use +
Frequency_ticks_on_person + Frequency_tick_check + TBD_concern, data=surv, na.action =
na.fail)
model_set21_test <- dredge(model21_test)
avg_model_set21_test <- model.avg(model_set21_test, subset = delta < 4)
car::S(avg_model_set21_test)
(ci <- confint(avg_model_set21_test, full=TRUE)) # create confidence intervals
exp(cbind(OR = coef(avg_model_set21_test, full=TRUE), ci)) # exponentiate
selected_model21 <- get.models(model_set21_test, subset = delta < 4)

## Anaplasma - total ticks
model22 <- glm(Pb_Ww_total_Ap_t ~ Gender + Age + Employment + Repellant_use +
Frequency_ticks_on_person + Frequency_tick_check + TBD_concern, data=surv)
car::S(model22) # summarize full model
(ci <- confint(model22)) # create confidence intervals
exp(cbind(OR = coef(model22), ci)) # exponentiate
step_model22 <- step(model22, direction="both") # stepwise model selection to determine
the best model to predict the risk of infected tick encounters
model22_best <- glm(Pb_Ww_total_Ap_t ~ Age + Frequency_tick_check, data=surv) # best model
as predicted by stepwise selection
car::S(model22_best) # summary of best model
(ci <- confint(model22_best)) # create confidence intervals
exp(cbind(OR = coef(model22_best), ci)) # exponentiate
ggeffects::ggpredict(model22_best, c("Frequency_tick_check", "Age"))
Ww_Ap_pp_effects <- plot(ggeffects::ggpredict(model22_best, c("Frequency_tick_check",
"Age")))

summary(surv$Pb_Ww_total_Ap_t)

# test with multi-model inference selection
model22_test <- glm(Pb_Ww_total_Ap_t ~ Gender + Age + Employment + Repellant_use +
Frequency_ticks_on_person + Frequency_tick_check + TBD_concern, data=surv, na.action =
na.fail)
model_set22_test <- dredge(model22_test)
avg_model_set22_test <- model.avg(model_set22_test, subset = delta < 4)
car::S(avg_model_set22_test)
(ci <- confint(avg_model_set22_test, full=TRUE)) # create confidence intervals
exp(cbind(OR = coef(avg_model_set22_test, full=TRUE), ci)) # exponentiate
selected_model22 <- get.models(model_set22_test, subset = delta < 4)
```

```
### all sites averaged into a single value for TOTAL ticks
## borrelia
model23 <- glm(Pb_all_total_Bb_t ~ Gender + Age + Employment + Repellant_use +
Frequency_ticks_on_person + Frequency_tick_check + TBD_concern, data=surv)
car::S(model23) # summarize full model
(ci <- confint(model23)) # create confidence intervals
exp(cbind(OR = coef(model23), ci)) # exponentiate
car::vif(model23)
ggeffects::ggpredict(model23, c("Frequency_tick_check", "Age"))

summary(surv$Pb_all_total_Bb_t)

## anaplasma
model24 <- glm(Pb_all_total_Ap_t ~ Gender + Age + Employment + Repellant_use +
Frequency_ticks_on_person + Frequency_tick_check + TBD_concern, data=surv)
car::S(model24) # summarize full model
(ci <- confint(model24)) # create confidence intervals
exp(cbind(OR = coef(model24), ci)) # exponentiate
```
